# Who is more likely to make causal claims in observational studies? The role of author experience, team size, cultural background, and gender in scientific framing

**DOI:** 10.1371/journal.pone.0354292

**Published:** 2026-08-12

**Authors:** Jun Wang, Bei Yu

**Affiliations:** 1 Independent Researcher, Syracuse, New York, United States of America; 2 School of Information Studies, Syracuse University, Syracuse, New York, United States of America; University of Texas Southwestern Medical Center at Dallas, UNITED STATES OF AMERICA

## Abstract

Scientific communication relies on language that conveys different levels of certainty about research findings. In observational studies, causal language—which attributes cause-and-effect relationships—is an important way researchers express certainty, as they must balance confidence in their findings against the limitations of observational data. Ideally, the use of causal language should depend only on the strength of the underlying evidence. However, through the analysis of over 90,000 abstracts from observational studies using computational linguistic and regression methods, we found that causal language is more common in work by less experienced authors, smaller research teams, male last authors, and researchers from countries with higher uncertainty avoidance indices—a cultural dimension reflecting a society’s preference for certainty over ambiguity. Our findings suggest that the use of causal language is not solely driven by the strength of evidence, but also by the sociocultural backgrounds of authors and their team composition. This work provides a new perspective for understanding systematic patterns in how scientists express certainty, emphasizing the importance of recognizing these human factors when evaluating scientific claims.

## Introduction

Scientific writing is fundamentally a social act [1]. The interpretation and communication of research findings may reflect not only the strength of the underlying evidence but also the sociocultural contexts in which researchers operate. Sociology of science scholarship posits that knowledge production is shaped by the context and identity of its producers [2], viewing objectivity as a collective property of scientific communities that emerges from shared standards and openness to critique [3]. Consequently, how scientists express certainty in their findings, including their choices to use causal versus correlational language, may be influenced by sociocultural factors beyond the strength of evidence alone.

This question of certainty expression is particularly important in observational research, where drawing causal conclusions has been a subject of ongoing debate in the scientific community. Observational studies serve as crucial sources of evidence when randomized controlled trials are infeasible or unethical. However, due to the limitations of observational data, drawing causal claims from observational evidence can raise concerns about overstated claims in the scientific literature, particularly when media coverage and press releases further amplify perceived certainty. Such concerns extend across various disciplines, including biomedicine [4–6], psychology [7,8], education [9,10], and social work [11].

Much of the current debate about causal language in observational research centers on whether such language appropriately reflects the strength of the underlying evidence. Our study shifts the focus to an additional dimension: the socio-cultural factors that may influence how scientists frame causal findings.

This shift in focus is motivated by two strands of prior work. First, studies of academic discourse have documented systematic differences in how writers express certainty: authors express confidence or caution through linguistic devices such as hedges and boosters [12], and these choices vary across languages and cultures [13,14] as well as between men and women [15]. If certainty expression in general is shaped by who the writer is, the choice between causal and correlational framing—a consequential form of certainty expression—may be as well. Second, advances in computational text analysis have made it possible to study such linguistic choices at scale, including classifiers that distinguish causal from correlational claims in scientific text [16–18].

Specifically, our goal was to examine whether characteristics of research teams and individual authors, including their country, gender, authorship position, team size, and publication history, correlate with the use of causal language when presenting conclusions in their structured abstracts.

In pursuit of this goal, we developed a transformer-based computational linguistic algorithm that is capable of differentiating between causal and correlational statements in biomedical domains. We applied it to 91,933 observational study abstracts and analyzed the results using logistic linear mixed-effects regression. In this context, we identified observational studies using metadata assigned by the NIH National Library of Medicine [19], which defines an observational study as “a work that reports on the results of a clinical study in which participants may receive diagnostic, therapeutic, or other types of interventions, but the investigator does not assign participants to specific interventions (as in an interventional study).”

Our analysis controlled for potential confounders including journal impact factor, study design, and a comprehensive set of over 1,400 Medical Subject Heading (MeSH) terms that together capture a wide range of research topics, methodologies, and key scientific concepts. Additionally, to account for journal-specific effects related to editorial policies and temporal trends, we modeled journal ISSN and publication year as random effects. After adjusting for these factors, we found that causal claims were more frequently used by less experienced authors, smaller teams, male last authors, and authors from countries with higher uncertainty avoidance indices—a cultural dimension reflecting a society’s preference for certainty over ambiguity [20].

## Materials and methods

### Regression model overview

In our regression model (see Table 1 for its formula), the dependent variable is whether the conclusion subsection of an article’s structured abstract contains one or more sentences with a causal statement. The primary explanatory variables include: (1) author writing experience, (2) author gender, (3) author country, and (4) team size. We will describe how these data were obtained and processed in the following subsections.

**Table 1:** The formula for the logistic linear mixed-effects regression model.

Has_a_Causal_Claim_in_the_Conclusion_Subsection_of_an_Abstract ∼		
// VARIABLES OF INTEREST		
	Num_coauthors	// log-scaled
+	Num_obs_studies_by_**first**_author	// log-scaled
+	Num_obs_studies_by_**last**_author	// log-scaled
+	Gender_of_**first**_author	// categorical: F, M, Unknown; reference: F
+	Gender_of_**last**_author	// categorical: F, M, Unknown; reference: F
+	Country_of_authors	// categorical: 40 countries and others; reference: others
// CONTROL VARIABLES		
+	Journal_rank	// log-scaled: SCImago journal rank
+	Num_obs_studies_in_the_journal	// log-scaled
+	$\sum_{i=1}^{N}{\text{MeSH\_term}}_{i}$	// binary: N = 1400 + terms; reference: absence of term
// RANDOM EFFECT		
+	(1 | Conclusion_length)	// categorical: the majority are 1, 2, 3, or 4 sentences
+	(1 | Journal_ISSN)	// categorical: 2,721 journals
+	(1 | Publication_year)	// categorical: the majority are from 2014 to 2025

The control variables include three parts: (1) journal rank (high-impact journals may have more restrictions on the use of causal language); (2) number of observational studies associated with a journal; (3) over 1400 MeSH terms, each appearing in at least 100 papers, representing various research designs, such as cross-sectional or cohort studies, as well as various topics and methods.

Our regression model is a mixed-effects logistic regression that includes three random effects: (1) *Conclusion Length*, measured by the number of sentences, to account for the greater likelihood of causal language in longer conclusions. This adjustment is necessary given that the dependent variable is defined as the presence of a causal statement in the conclusion of an observational study abstract. In our dataset, 99% of conclusions contain four or fewer sentences. (2) *Journal ISSN*, to capture variations in editorial guidelines and practices regarding causal language across publications. (3) *Publication Year*, to control for temporal shifts in language use and evolving academic norms in response to changing policies and cultural trends [21].

### Obtaining observational studies

**Fig 1:**
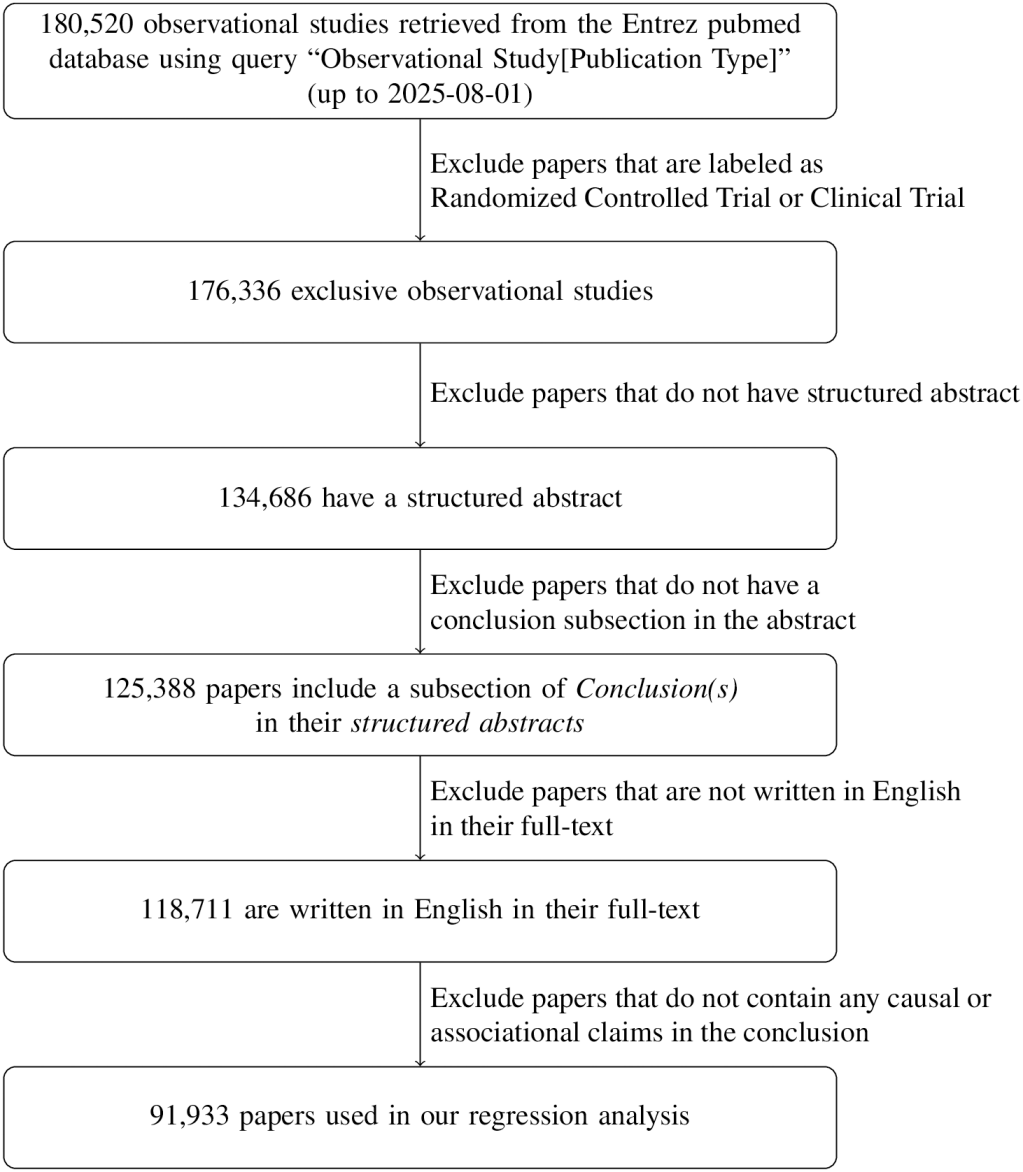
Flowchart of the process for collecting observational studies for our analysis.

We queried the PubMed database using the search term “Observational Study[Publication Type]” and retrieved over 180,000 papers, most of which were published in 2014 or later following the introduction of the MeSH term “Observational Study” in 2014. After removing records that were also labeled as randomized controlled trials or clinical trials, 176,336 exclusive observational studies remained. We then restricted the dataset to studies with structured abstracts (134,686 papers), and further to those containing a clearly defined conclusion subsection (125,388 papers). Limiting to studies whose full texts are written in English yielded 118,711 papers. Finally, we excluded papers whose conclusions did not contain any causal or associational claims—typically those limited to descriptive findings, recommendations, study implications, or directions for future work—resulting in a final dataset of 91,933 observational studies used in our regression analysis. See Fig 1 for the flowchart of the process.

### Identifying causal and correlational claims

In our regression analysis, we defined the dependent variable as the presence or absence of a causal statement in the conclusion of an observational study abstract. Specifically, a conclusion is considered causal if it includes at least one sentence with a causal statement.

To identify whether a sentence has a causal statement, we used the following scheme that is based on the one introduced in [4] and [16]:

- A *causal* statement uses terms indicating direct causation (e.g., *increase, make, lead to, effective in, contribute to*) or uses the modal verb *can* followed by a causal verb.
- A *correlational* statement is either a common *correlational* statement that employs language suggesting an association (e.g., *associated with, predictor, linked with*), or a *conditional causal* statement that uses qualifiers (e.g., *may, might, appear to, probably*) to tone down the level of certainty. Conditional causal statements are classified as correlational, following prior research showing that general readers often struggle to distinguish between the two [8].
- Neither causal nor correlational.

To automatically classify sentences as causal, correlational, or neither, we annotated a corpus of 3,061 PubMed research conclusion sentences [17]. Using this corpus, we trained a causal language prediction model based on BioBERT [22], achieving a macro-F1 score of 0.89 under 5-fold cross-validation. Empirical comparisons with the ChatGPT family of models further confirmed that our task-specific model retains a performance advantage on this task [23,24]. Fine-tuned transformer models have been shown to outperform other methods for identifying causal claims in scientific text, with strong results demonstrated in both biomedical and social science domains [17,25].

Of the 91,933 observational study abstracts that contain causal or correlational statements, we found that 31.7% had causal claims. For comparison, a similar proportion (31%) was reported by [26], who manually analyzed 525 peer-reviewed papers in the fields of obesity and nutrition.

### Author gender

We inferred gender from each author’s forename, categorizing names as male, female, or unknown. The unknown category included cases where a given name was missing (e.g., only initials provided) or where the gender inference algorithm returned a low-confidence result. About 60% of the unknown cases were due to the latter, a challenge particularly common for names without clear gender markers, such as many Chinese and Korean names.

Using a dataset of 6 million (name, gender) pairs from WikiData, we developed and open-sourced a name-to-gender inference algorithm [27] based on LightGBM, a gradient boosting method [28]. The algorithm uses position-specific characters from both the beginning and the end of each forename, achieving performance comparable to the best of the five name-to-gender inference tools (four of them require a paid service for bulk access) when evaluated on a benchmark dataset [29].

In our implementation, a name is labeled as male if its male confidence exceeds 0.82 and as female if its female confidence exceeds 0.78; otherwise, it is classified as gender-unknown. These thresholds were determined through searching a grid of possible values for best F1-score on the benchmark dataset under the following constraint: at most 12.5% of names are assigned to gender-unknown (this setting allows for about 98% precision and 96% recall for both genders). The lower female threshold (0.78 for female vs 0.82 for male) compensates for the training data’s 75% male majority, which biases predictions toward male.

**Fig 2:**
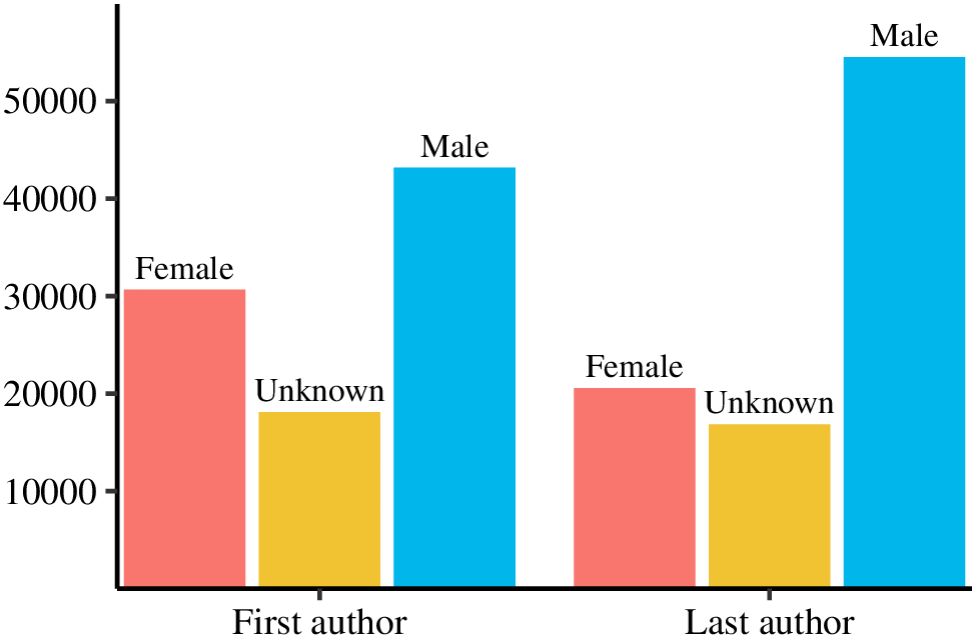
Gender distribution of first and last author.

As shown in Fig 2, the male-to-female ratio for first authors is 1.4:1, while for last authors, it increases to 2.7:1—nearly doubling the ratio for first authors. This pattern, where male authors outnumber female authors in both roles, aligns with previous findings on gender imbalances in biomedical research publications [30], with a more noticeable difference observed among last authors.

Following previous research on authorship in biomedical fields [31], we only consider first and last author, as these positions in biomedical research typically represent the primary contributors: first authors are usually those who contributed most substantially to the writing, while last authors are the senior or principal investigators overseeing the project.

### Author country

Author affiliations in the PubMed metadata provide a basis for inferring country information. However, this can be challenging when the country is not explicitly mentioned. To address the challenge, we developed a LinearSVC-based text classification model to infer the country associated with an author’s affiliation, leveraging a training dataset of 2.7 million organizations with known country information from the ORCID database [32].

We evaluated the algorithm on 1,000 randomly sampled affiliations from the PubMed metadata, which we divided into two groups: (1) affiliations without necessary information to infer the country (e.g., affiliation listed simply as “Division of Infectious Diseases”) which include 7 cases; and (2) affiliations with sufficient information to infer the country which include 993 cases. For the 7 cases in Group 1, the algorithm appropriately returned low confidence scores (below 0.8). In contrast, for all cases in Group 2, the algorithm produced accurate country inferences. For additional details, please visit our GitHub page [33].

**Fig 3:**
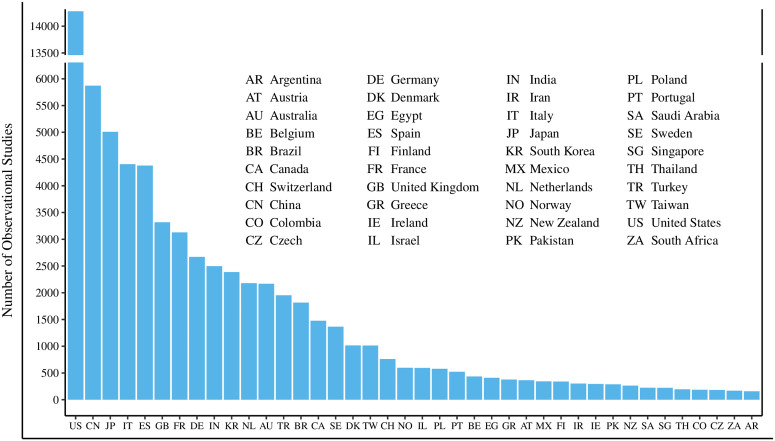
Distribution of author country.

In our study, each paper is assigned to one of 40 countries or *others*: we consider only countries with at least 150 observational studies in our dataset, totaling 40 countries (Fig 3). The *others* category, used as the reference in our regression analysis, includes 23,204 observational studies, broken down as follows: (1) 18,399 from multiple countries, (2) 2,336 from various other countries (each contributing fewer than 150 studies), (3) 1,636 with missing affiliation data, and (4) 833 with low-confidence classification (confidence score below 0.8).

### Author writing experience and name disambiguation

An author’s writing experience is measured by the number of observational studies the author is associated with in our dataset. It is challenging to determine which publications belong to which real-world authors, especially when common names are shared by different authors or when the same author publishes under different names. To disambiguate author names, we used the Semantic Scholar API [34] to obtain a unique ID for each author per article. Author IDs were successfully retrieved for 99.8% of the authors; for the remainder, a unique random ID was generated and assigned.

Semantic Scholar’s system of author name disambiguation, called S2AND (Semantic Scholar Author Name Disambiguation), works by first grouping papers that share the same or lexically similar author name strings, then using supervised learning to predict the likelihood that pairs of papers were written by the same individual based on features such as co-authors, affiliations, titles, venues, and abstracts, and finally applying unsupervised clustering to assign papers to distinct author identities based on the predicted pairwise similarities. S2AND has demonstrated performance comparable to state-of-the-art methods across eight benchmark datasets [35].

**Fig 4:**
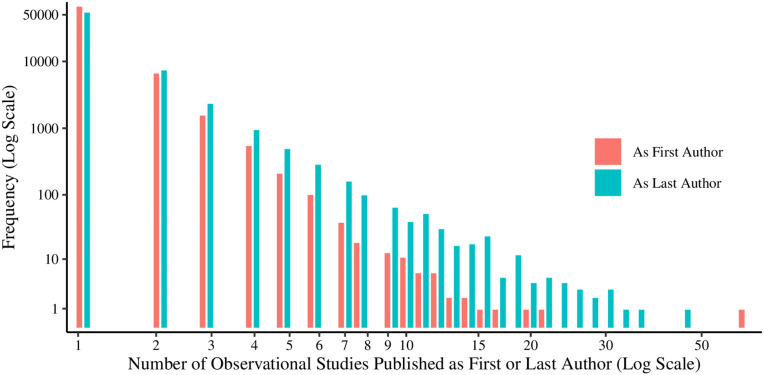
Number of papers by author (author writing experience).

Fig 4 shows the distribution of the number of observational studies published by authors in either the first or last author position, with both axes on a log scale. The x-axis represents the number of papers an author has published in either role, while the y-axis indicates the frequency of authors with that publication count. The distribution follows a heavy-tailed pattern, where most authors have published only a few papers, while a relatively small group of authors has a significantly higher publication count.

### Team size

**Fig 5:**
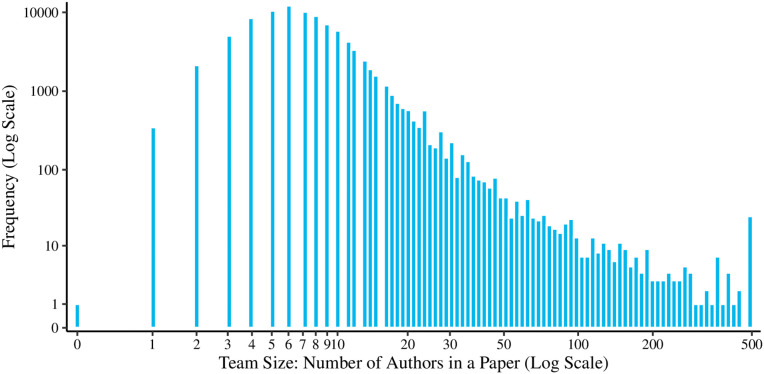
Number of authors per paper. The 500-author ceiling seen in some papers is simply a byproduct of our data collection process: PubMed and Semantic Scholar allowed us to retrieve no more than 500 authors per paper.

Fig 5 presents the distribution of team sizes, measured by the number of authors per paper on a log scale for both the x-axis and y-axis. The x-axis represents the number of authors per paper, ranging from single-author papers to large collaborative teams exceeding 500 authors (as we can only retrieve up to 500 authors by combining PubMed and the Semantic Scholar data). The y-axis denotes the frequency of papers with a given team size, also on a log scale. The distribution exhibits a right-skewed pattern, indicating that most papers have relatively small author teams, with the highest frequencies occurring for papers with around 6 authors.

### Journal rank

**Fig 6:**
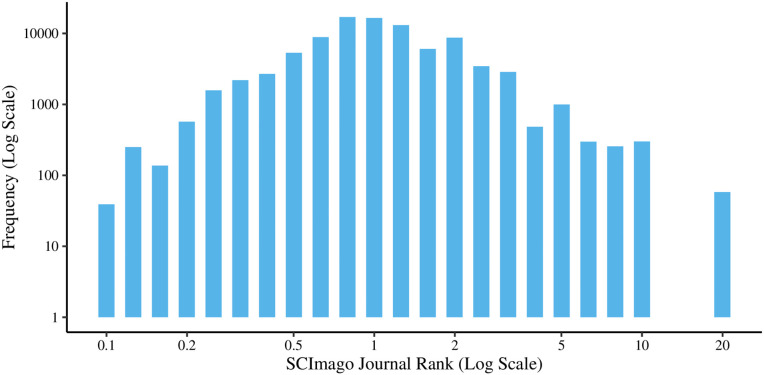
Journal rank distribution.

We downloaded publicly accessible SCIMago Journal Rank (SJR) data for 2024 and prior years [36]. For journals in our dataset that could not be matched to the 2024 data via their ISSN, we attempted to retrieve their information from previous years. If a journal remained unmatched (which occurred for only 0.35% of the papers), we assigned it an SJR score of 0.49, corresponding to the bottom quartile in our list of 2,721 journals, based on the assumption that journals not listed in the SJR database are likely to have a low rank. As shown in Fig 6, the distribution exhibits strong right-skewness, indicating that while most journals have relatively low SJR values, a small number achieve substantially higher rankings.

## Results and discussion

Our mixed-effects logistic regression model examined predictors of causal language use in the conclusion subsections of observational study abstracts. The model included author country, gender, authorship position, writing experience, team size, journal characteristics, and over 1,400 MeSH terms as fixed effects, with journal ISSN, publication year, and conclusion length specified as random effects. We used the *glmer* function from the R package *lme*4 [37] on the 91,933 observations to run the analysis.

We report the estimated effects in three tables organized by variable type: author country (Table 2), author-level characteristics (Table 3), and control variables (Table 4). In the tables, each row reports the estimated regression coefficient (β) for one predictor, with its standard error in parentheses and significance stars. Because the model is a logistic regression, each coefficient is on the log-odds scale: a positive β indicates higher odds of using causal language, and a negative β indicates lower odds, each measured relative to a reference (baseline) category. Every categorical predictor is estimated against an explicitly labeled reference: *Women* for author gender, the pooled *Others* group for author country, and the absence of the term for study design and the MeSH terms. The reference category has a coefficient of zero by construction and is therefore displayed as “0.000 (reference)” rather than as an estimated value.

**Table 2:** Estimated effects of author country on the likelihood of using causal language in the conclusion section of observational study abstracts. Coefficients (β) are on the log-odds scale, with standard errors in parentheses. ^***^p<0.001; ^**^p<0.01; ^*^p<0.05.

Country	β	(SE)		Country	β	(SE)	
Others (reference): β=0.000 (baseline for all countries)							
New Zealand	−0.354	(0.151)	^*^	Egypt	0.047	(0.114)	
United States	−0.308	(0.027)	^***^	China	0.050	(0.037)	
Norway	−0.298	(0.101)	^**^	France	0.055	(0.044)	
Denmark	−0.274	(0.080)	^***^	Ireland	0.062	(0.132)	
Canada	−0.270	(0.068)	^***^	Brazil	0.072	(0.059)	
South Africa	−0.186	(0.180)		Portugal	0.117	(0.101)	
Sweden	−0.162	(0.068)	^*^	Netherlands	0.122	(0.051)	^*^
Israel	−0.137	(0.099)		Spain	0.141	(0.039)	^***^
Australia	−0.125	(0.054)	^*^	Colombia	0.174	(0.161)	
United Kingdom	−0.119	(0.045)	^**^	Czech	0.179	(0.165)	
Finland	−0.088	(0.130)		Switzerland	0.184	(0.083)	^*^
Argentina	−0.086	(0.184)		Thailand	0.198	(0.161)	
Iran	−0.080	(0.134)		India	0.202	(0.051)	^***^
Japan	−0.068	(0.040)		Belgium	0.215	(0.108)	^*^
Mexico	−0.042	(0.127)		Germany	0.225	(0.046)	^***^
Singapore	−0.038	(0.158)		Greece	0.250	(0.117)	^*^
South Korea	−0.023	(0.054)		Poland	0.300	(0.096)	^**^
Taiwan	−0.022	(0.078)		Italy	0.329	(0.038)	^***^
Saudi Arabia	−0.010	(0.152)		Austria	0.346	(0.123)	^**^
Turkey	0.032	(0.057)		Pakistan	0.350	(0.142)	^*^

**Table 3:** Estimated effects of author-level characteristics—gender, writing experience (number of observational studies published), and team size—on the likelihood of using causal language. ^***^p<0.001; ^**^p<0.01; ^*^p<0.05^.^

Author writing experience (num of obs. studies) (log-scaled)			
As first author	−0.119	(0.028)	^***^
As last author	−0.167	(0.018)	^***^
**Team size** (log-scaled)			
Number of co-authors	−0.089	(0.017)	^***^
**First author gender**			
Women (reference)	0.000		
Men	0.010	(0.018)	
Unknown	0.033	(0.025)	
**Last author gender**			
Women (reference)	0.000		
Men	0.057	(0.020)	^**^
Unknown	0.032	(0.028)	

**Table 4:** Estimated effects of various control variables. For study design variables (represented by specific MeSH terms) and other MeSH terms, the reference category corresponds to the absence of the respective term. ^***^p<0.001; ^**^p<0.01; ^*^p<0.05^.^

Journal (log-scaled)			
SCIMago journal rank	−0.091	(0.032)	^**^
Observational studies published	−0.042	(0.009)	^***^
**Study design**			
Cross-Sectional	−0.339	(0.028)	^***^
Case-Control	−0.278	(0.043)	^***^
Longitudinal	−0.161	(0.040)	^***^
Cohort	−0.161	(0.028)	^***^
Retrospective	−0.022	(0.020)	
Prospective	0.026	(0.019)	
Follow-Up	0.063	(0.030)	^*^
**Other terms beyond study design**			
1400 + MeSH terms (see S1 Table), each associated with 100 or more papers			

### Author country

Author country emerged as one of the most prominent structural predictors in the model. As shown in Table 2, researchers affiliated with eight English-speaking and Nordic countries were significantly less likely to use causal language in their abstract conclusions.

Specifically, authors from New Zealand (β=−0.354, *p* < .05), the United States (β=−0.308, *p* < .001), Norway (β=−0.298, *p* < .01), Denmark (β=−0.274, *p* < .001), Canada (β=−0.270, *p* < .001), Sweden (β=−0.162, *p* < .05), Australia (β=−0.125, *p* < .05), and the United Kingdom (β=−0.119, *p* < .01) all showed significantly lower odds of causal language relative to the reference category (all publications excluding those authored solely by the top 40 countries). By contrast, several Continental European and South Asian countries were associated with significantly higher odds, including Italy (β=0.329, *p* < .001), Austria (β=0.346, *p* < .01), Pakistan (β=0.350, *p* < .05), Germany (β=0.225, *p* < .001), Poland (β=0.300, *p* < .01), India (β=0.202, *p* < .001), and Spain (β=0.141, *p* < .001). A large cluster of countries—including China, Japan, South Korea, Iran, Turkey, and Egypt—did not differ significantly from the reference baseline.

These coefficients can be read directly as odds ratios by exponentiation. For example, New Zealand, with the most negative estimate (β=−0.354), corresponds to an odds ratio of $e^{-0.354}\approx 0.70$—about 30% lower odds of using causal language than the reference group—while Pakistan, near the opposite extreme (β=0.350), corresponds to $e^{0.350}\approx 1.42$, or about 42% higher odds. In each case the reported *p*-value tests whether a country differs from the pooled *Others* group (papers with authors from multiple countries, from countries with fewer than 150 studies, or with missing or low-confidence affiliations).

**Fig 7:**
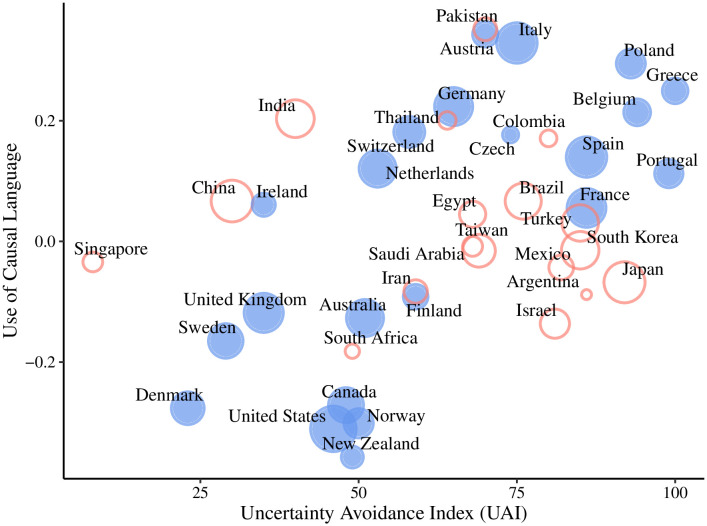
Authors from countries with higher uncertainty avoidance index scores—reflecting a greater cultural preference for certainty—tend to use causal language more frequently. The correlation is moderate across all 40 countries (Pearson’s *r* = 0.45, *p* < 0.01), and strengthens significantly among 22 Western countries (*r* = 0.70, *p* < 0.001), defined here as European nations along with United States, Canada, Australia and New Zealand, based on Geert Hofstede’s country-region classification (see Hofstede, 2010, Table 6.1) [20]. The size of the circles corresponds to the log scale of the number of observational studies published under the country’s name.

Beyond these country-specific estimates, a broader regional pattern emerges. As shown in Fig 7, the results suggest systematic cross-country variation, pointing to a cultural-level explanation for the observed differences. Countries with higher scores on Hofstede’s Uncertainty Avoidance Index (UAI)—reflecting a greater societal preference for certainty—tended to exhibit significantly higher rates of causal language use (Pearson’s r=0.45, *p* < .01, across all 40 countries). This observation is consistent with existing literature on cultural influences in scientific communication: for example, French researchers use fewer expressions of uncertainty than English or Norwegian counterparts [13], and German texts favor stronger modal expressions (e.g., *must* and *will*) that convey necessity or certainty, whereas English more often uses possibility markers [14]. This correlation became notably stronger when the sample was restricted to 22 Western countries (r=0.70, *p* < .001), defined as European nations along with United States, Canada, Australia and New Zealand, following Geert Hofstede’s country-region classification (see Hofstede, 2010, Table 6.1) [20]. This pattern suggests that, within culturally comparable countries, the tendency to use causal language may be more strongly associated with national norms of uncertainty avoidance.

Taken together, these results suggest that the tendency to use causal language in observational study conclusions reflects not only individual-level variation in methodological training, but also broader cultural dispositions toward certainty. English-speaking and Nordic scientific communities tend to score relatively low on the Uncertainty Avoidance Index (UAI) and show patterns consistent with more cautious use of causal language. In contrast, researchers from higher-UAI countries may face institutional or social expectations that favor language that presents findings as clear and definitive outcomes.

It is worth noting, however, that UAI as a single-dimensional metric has recognized limitations as a measure of cross-cultural differences in communication [38]. Countries with similar UAI scores may differ substantially in their linguistic practices for reasons the index does not capture, and the notably stronger correlation observed within European countries and their English-speaking counterparts compared to all 40 countries suggests that UAI’s explanatory power may be more salient in Western cultural contexts than in others. Future work incorporating additional cultural metrics would help to better characterize the full diversity of national norms governing scientific language use beyond the Western context.

### Author writing experience

Beyond geography, several author-level characteristics were independently associated with causal language use. Table 3 shows that both first-author and last-author publication experience — operationalized as the log-scaled number of observational studies published — were negatively associated with causal language (first author: β=−0.119, *p* < .001; last author: β=−0.167, *p* < .001), suggesting that more experienced researchers tend to frame findings more cautiously. One plausible interpretation is that researchers with more publications have had greater exposure to disciplinary expectations around causal language, leading them to express findings with greater restraint [39]. The stronger effect observed for last authors—typically senior investigators responsible for overseeing the study and shaping the final manuscript—further underscores this pattern, as senior authors are especially well positioned to influence how conclusions are worded through both direct editing and mentorship.

At the same time, alternative explanations are worth considering. Less experienced researchers may simply be less familiar with these conventions, leading to more frequent use of causal language. Conversely, they may have greater familiarity with newer causal inference methods and thus feel more confident in making stronger claims. Nonetheless, the consistent negative association across both authorship positions suggests that author seniority plays an important role in shaping how findings from observational studies are communicated.

### Team size

Team size was negatively associated with causal language use (β=−0.089, *p* < .001), indicating that larger teams produce more cautious abstracts. This pattern is consistent with a collective deliberation effect, whereby multiple co-authors—often bringing diverse disciplinary backgrounds and methodological expertise—are more likely to identify and moderate overly strong causal claims during drafting and revision. This interpretation aligns with Hyland’s observation that scientific writing involves negotiating meaning through shared rhetorical conventions [12]; in collaborative contexts, such negotiation occurs among co-authors as well, fostering more carefully calibrated claims and reducing the likelihood of overstatement.

### Author gender

With respect to author gender, first-author gender was not significantly associated with causal language use. However, papers whose last author was identified as male showed a small but statistically significant positive association (β=0.057, *p* < .01), indicating slightly higher odds of causal language when the senior author was male, relative to female senior authors. Because last authors typically serve as senior investigators who shape the framing of a manuscript, this pattern may reflect gendered linguistic tendencies, differences in academic seniority, or both.

Prior research on gendered linguistic tendencies offers one possible explanation. Male authors have been found to use more linguistic boosters—expressions that intensify or strengthen claims (e.g., *clearly*)—which may contribute to a more assertive presentation of results [40,41]. Female authors, by contrast, tend to use more hedging language (e.g., *may*), signaling greater caution in expressing claims, a pattern supported by a meta-analysis finding that women are, on average, more likely to adopt tentative language [15].

### Study design

Study design was included in the model as a set of binary control variables representing different study types, and their associations with causal language were consistent with the hierarchy of evidence [42]. In Table 4, cross-sectional and case-control studies showed the largest negative associations (β=−0.339 and β=−0.278, respectively, both *p* < .001), followed by longitudinal and cohort designs (β=−0.161 for both, *p* < .001). Follow-up studies showed a small positive association (β=0.063, *p* < .05). That weaker designs were associated with more cautious framing, and stronger designs with more assertive framing, is consistent with methodological expectations and supports the validity of the model specification.

### Journal characteristics

Journal characteristics were also included as controls in the model. Both SCIMago journal rank (β=−0.091, *p* < .01) and the number of observational studies previously published in the journal (β=−0.042, *p* < .001) showed negative associations (both log-scaled), indicating that higher-ranked journals that publish more observational studies tend to feature more cautiously framed abstracts. This pattern is consistent with editorial norms that emphasize restrained interpretation of non-experimental findings, and aligns with expectations for how editorial standards shape observational reporting.

### Limitations

The following limitations of this study warrant acknowledgment. First, this is itself an observational study of observational studies, and the regression coefficients reported here should be interpreted as associations rather than causal effects.

Second, our analysis focused on the conclusion sentences of abstracts, which may not fully reflect how authors frame causal claims in the main text.

Third, author country was assigned based on institutional affiliation rather than researcher nationality or training, which may introduce misclassification for researchers who have moved between countries.

Fourth, our findings are drawn from English-language biomedical observational studies indexed by the National Library of Medicine, and may not generalize to other scientific domains or publication cultures.

Fifth, because the analysis includes only English-language studies and English is not the first language of many authors, the observed differences in causal language use cannot be fully attributed to cultural influences: they may also reflect how authors write in English as a second language, and the two influences are inherently intertwined.

Finally, the country-level patterns observed in our analysis reflect a broad association between cultural context and causal language use, and should not be interpreted as evidence that any individual researcher writes in a particular way because of their cultural background. Nor should they be taken as a judgment on the scientific quality of researchers from any specific country. Cultural factors tend to shape behavior in general ways, and there is considerable variation among researchers within every country.

## Conclusions

Our findings suggest that the use of causal language in observational research abstracts is shaped not only by the strength of the underlying evidence, but also by who is doing the research and the context in which it is conducted. Researchers from certain countries, those with less publishing experience, those working in smaller teams, and those with male last authors were more likely to use causal language. These patterns suggest that how scientists communicate their findings may be influenced by cultural background, professional experience, and team composition—not just by the quality of the evidence itself.

These findings point to several directions worth exploring. Supporting early-career researchers in developing awareness of language norms in scientific writing, encouraging collaborative peer review, and providing clearer author guidelines on causal language in journal submission portals are all potential avenues for helping align the language used in research abstracts with what the evidence can and cannot support. The fact that more experienced authors, larger teams, and higher-ranked journals were associated with more cautious language further suggests that collaborative and deliberative research environments may also contribute to careful scientific communication, alongside methodological training.

More broadly, this work highlights that the way scientists express certainty in their findings is not a purely technical decision. It is also shaped by human and institutional factors that are often invisible in the final published paper. Recognizing these influences is an important step toward more transparent and trustworthy scientific communication.

## Supporting information

S1 Table Estimated effects of more than 1,400 MeSH terms. This table reports the estimated effects of more than 1,400 MeSH terms included as control variables in the regression model. Each MeSH term is represented by a binary indicator, with the absence of the term serving as the reference category. A MeSH term was included if it was assigned to at least 100 articles in the dataset and was not a country name, because author country was analyzed separately.
(PDF)

## References

[1] Bazerman C. Scientific Writing as a Social Act: A Review of the Literature of the Sociology of Science. New Essays in Technical and Scientific Communication: Research, Theory, Practice. Baywood Publishing Company, Inc. 1983. 10.2190/netc8

[2] Haraway D. Situated Knowledges: The Science Question in Feminism and the Privilege of Partial Perspective. Feminist Studies. 1988;14(3):575. doi: 10.2307/3178066

[3] Xia X, Ouellet M, Patankar SP, Tamir DI, Bassett DS. Citation Sentiment Reflects Multiscale Sociocultural Norms. In: 2024. https://arxiv.org/abs/2411.09675

[4] Sumner P, Vivian-Griffiths S, Boivin J, Williams A, Venetis CA, Davies A, et al. The association between exaggeration in health related science news and academic press releases: retrospective observational study. BMJ. 2014;349:g7015. doi: 10.1136/bmj.g7015 25498121 PMC4262123

[5] Hernán MA. The C-Word: Scientific Euphemisms Do Not Improve Causal Inference From Observational Data. Am J Public Health. 2018;108(5):616–9. doi: 10.2105/AJPH.2018.304337 29565659 PMC5888052

[6] Olarte Parra C, Bertizzolo L, Schroter S, Dechartres A, Goetghebeur E. Consistency of causal claims in observational studies: a review of papers published in a general medical journal. BMJ Open. 2021;11(5):e043339. doi: 10.1136/bmjopen-2020-043339 34016660 PMC8141434

[7] Bleske-Rechek A, Morrison KM, Heidtke LD. Causal inference from descriptions of experimental and non-experimental research: public understanding of correlation-versus-causation. J Gen Psychol. 2015;142(1):48–70. doi: 10.1080/00221309.2014.977216 25539186

[8] Adams RC, Sumner P, Vivian-Griffiths S, Barrington A, Williams A, Boivin J, et al. How readers understand causal and correlational expressions used in news headlines. J Exp Psychol Appl. 2017;23(1):1–14. doi: 10.1037/xap0000100 27808530

[9] Morrison K, van der Werf G. Searching for causality in educational research. Educational Research and Evaluation. 2016;22(1–2):1–5. doi: 10.1080/13803611.2016.1195081

[10] Adlakha V, Kuo E. Critical issues in statistical causal inference for observational physics education research. Phys Rev Phys Educ Res. 2023;19(2). doi: 10.1103/physrevphyseducres.19.020160

[11] Rubin A, Parrish D. Problematic phrases in the conclusions of published outcome studies: Implications for evidence-based practice. Research on Social Work Practice. 2007;17(3):334–47.

[12] Hyland K. Disciplinary discourses: social interactions in academic writing. University of Michigan Press. 2004.

[13] Vold ET. Epistemic modality markers in research articles: a cross‐linguistic and cross‐disciplinary study. Int J App Linguistics. 2006;16(1):61–87. doi: 10.1111/j.1473-4192.2006.00106.x

[14] Kranich S. To hedge or not to hedge: the use of epistemic modal expressions in popular science in English texts, English–German translations, and German original texts. Text & Talk - An Interdisciplinary Journal of Language, Discourse & Communication Studies. 2011;31(1):77–99. doi: 10.1515/text.2011.004

[15] Leaper C, Robnett RD. Women are more likely than men to use tentative language, aren’t they? A meta-analysis testing for gender differences and moderators. Psychology of Women Quarterly. 2011;35(1):129–42.

[16] LI Y, Zhang J, Yu B. An NLP Analysis of Exaggerated Claims in Science News. In: Proceedings of the 2017 EMNLP Workshop: Natural Language Processing meets Journalism, 2017. 106–11. 10.18653/v1/w17-4219

[17] Yu B, Li Y, Wang J. Detecting Causal Language Use in Science Findings. In: Proceedings of the 2019 Conference on Empirical Methods in Natural Language Processing and the 9th International Joint Conference on Natural Language Processing (EMNLP-IJCNLP), 2019. 4663–73. 10.18653/v1/d19-1473

[18] Yu B, Wang J, Guo L, Li Y. Measuring Correlation-to-Causation Exaggeration in Press Releases. In: Proceedings of the 28th International Conference on Computational Linguistics, 2020. 4860–72. 10.18653/v1/2020.coling-main.427

[19] NIH National Library of Medicine. Medical Subject Headings (MeSH); 2014. Visited 2025 February 13. https://www.ncbi.nlm.nih.gov/mesh/68064888

[20] Hofstede G, Hofstede GJ, Minkov M. Cultures and Organizations: Software of the Mind. 3rd ed. New York: McGraw-Hill. 2010.

[21] Hyland K, Jiang F. Academic Discourse and Global Publishing: Disciplinary Persuasion in Changing Times. Routledge. 2019.

[22] Lee J, Yoon W, Kim S, Kim D, Kim S, So CH, et al. BioBERT: a pre-trained biomedical language representation model for biomedical text mining. Bioinformatics. 2020;36(4):1234–40. doi: 10.1093/bioinformatics/btz682 31501885 PMC7703786

[23] Kim Y, Guo L, Yu B, Li Y. Can ChatGPT Understand Causal Language in Science Claims?. In: Proceedings of the 13th Workshop on Computational Approaches to Subjectivity, Sentiment, & Social Media Analysis, 2023. 379–89.

[24] Chen S, Li Y, Lu S, Van H, Aerts HJWL, Savova GK, et al. Evaluating the ChatGPT family of models for biomedical reasoning and classification. J Am Med Inform Assoc. 2024;31(4):940–8. doi: 10.1093/jamia/ocad256 38261400 PMC10990500

[25] Norouzi R, Kleinberg B, Vermunt JK, van Lissa CJ. Capturing causal claims: A fine-tuned text mining model for extracting causal sentences from social science papers. Res Synth Methods. 2025;16(1):139–56. doi: 10.1017/rsm.2024.13 41626898 PMC12621500

[26] Cofield SS, Corona RV, Allison DB. Use of causal language in observational studies of obesity and nutrition. Obes Facts. 2010;3(6):353–6. doi: 10.1159/000322940 21196788 PMC3280017

[27] Wang J. High-accuracy name-based gender prediction using LightGBM: a simple approach across global datasets. https://github.com/junwang4/name-to-gender-inference. 2024.

[28] Ke G, Meng Q, Finley T, Wang T, Chen W, Ma W. LightGBM: A Highly Efficient Gradient Boosting Decision Tree. In: Neural Information Processing Systems, 2017. 3146–54.

[29] Santamaría L, Mihaljević H. Comparison and benchmark of name-to-gender inference services. PeerJ Comput Sci. 2018;4:e156. doi: 10.7717/peerj-cs.156 33816809 PMC7924484

[30] Ioannidis JPA, Boyack KW, Collins TA, Baas J. Gender imbalances among top-cited scientists across scientific disciplines over time through the analysis of nearly 5.8 million authors. PLoS Biol. 2023;21(11):e3002385. doi: 10.1371/journal.pbio.3002385 37988334 PMC10662734

[31] Lerchenmueller MJ, Sorenson O, Jena AB. Gender differences in how scientists present the importance of their research: observational study. BMJ. 2019;367:l6573. doi: 10.1136/bmj.l6573 31843745 PMC7190066

[32] How do I get the public data file?. Visited 2026 July 13. https://support.orcid.org/hc/en-us/articles/360006897394-How-do-I-get-the-public-data-file

[33] Wang J. Inferring Countries from Publication Affiliations. https://github.com/junwang4/affiliation-to-country-inference

[34] Semantic Scholar API. Visited 2026 July 13. https://api.semanticscholar.org/ 2023.

[35] Subramanian S, King D, Downey D, Feldman S. S2AND: A Benchmark and Evaluation System for Author Name Disambiguation. In: 2021 ACM/IEEE Joint Conference on Digital Libraries (JCDL), 2021. 170–9. 10.1109/jcdl52503.2021.00029

[36] SCImago. SJR–SCImago Journal & Country Rank. Visited 2026 July 13. https://www.scimagojr.com/journalrank.php

[37] Bates D, Mächler M, Bolker B, Walker S. Fitting linear mixed-effects models using lme4. Journal of Statistical Software. 2015;67:1–48. doi: 10.18637/jss.v067.i01

[38] McSweeney B. The Essentials of Scholarship: A Reply to Geert Hofstede. Human Relations. 2002;55(11):1363–72. doi: 10.1177/00187267025511005

[39] Hyland K. Writing in the disciplines: Research evidence for specificity. Taiwan International ESP Journal. 2009;1(1):5–22.

[40] Tannen D. The power of talk: Who gets heard and why. Harvard Business Review. 1995;73(5):138–48.

[41] Nasri M, Biria R, Karimi M. Projecting Gender Identity in Argumentative Written Discourse. Int J Appl Linguist Engl Lit. 2018;7(3):201. doi: 10.7575/aiac.ijalel.v.7n.3p.201

[42] Murad MH, Asi N, Alsawas M, Alahdab F. New evidence pyramid. Evid Based Med. 2016;21(4):125–7. doi: 10.1136/ebmed-2016-110401 27339128 PMC4975798

