# Supplementary material for: Who is more likely to make causal claims in observational studies? The role of author experience, team size, cultural background, and gender in scientific framing
Source: PLoS One. 2026 Aug 12;21(8):e0354292. doi: 10.1371/journal.pone.0354292 (PMC13466022; doi:10.1371/journal.pone.0354292)
Supplement: Supplementary file 1 [file pone.0354292.s001.pdf]

S1 Table. Estimated effects of more than 1,400 MeSH terms.

Coronary Artery Disease/etiology	-1.175 (0.388)**
Femoral Artery/diagnostic imaging	-0.910 (0.287)**
Bacteremia/epidemiology	-0.895 (0.249)***
Wet Macular Degeneration/drug therapy	-0.842 (0.330)*
Lymphocytes	-0.814 (0.396)*
Biomarkers	-0.807 (0.104)***
Cardiovascular Diseases/complications	-0.786 (0.276)**
Pain, Postoperative/epidemiology	-0.785 (0.238)***
Sex Characteristics	-0.727 (0.210)***
Biomarkers, Tumor/metabolism	-0.724 (0.244)**
Cardiopulmonary Bypass/adverse effects	-0.720 (0.246)**
Heart Arrest/mortality	-0.708 (0.260)**
Fluorodeoxyglucose F18	-0.704 (0.341)*
Geriatric Assessment	-0.695 (0.161)***
Myocardium/pathology	-0.679 (0.290)*
Biomarkers, Tumor/blood	-0.679 (0.213)**
Sleep Wake Disorders/epidemiology	-0.654 (0.254)*
Kidney Failure, Chronic/physiopathology	-0.647 (0.291)*
Enzyme-Linked Immunosorbent Assay	-0.639 (0.187)***
Pneumonia, Viral/epidemiology	-0.631 (0.445)
Polymorphism, Single Nucleotide	-0.613 (0.155)***
Pre-Eclampsia/epidemiology	-0.603 (0.283)*
Vitamin D/blood	-0.599 (0.263)*
Transcatheter Aortic Valve Replacement/mortality	-0.596 (0.328)
Nutrition Surveys	-0.594 (0.196)**
Black or African American/statistics & numerical data	-0.585 (0.342)
Glasgow Coma Scale	-0.575 (0.164)***
Arrhythmias, Cardiac/diagnosis	-0.569 (0.279)*
Hypotension/etiology	-0.568 (0.250)*
Ventricular Dysfunction, Left/diagnostic imaging	-0.567 (0.250)*
Cytokines/blood	-0.562 (0.210)**
Postoperative Complications/blood	-0.561 (0.231)*
Hydrogen-Ion Concentration	-0.559 (0.255)*
Choroid/blood supply	-0.556 (0.272)*
Carotid Intima-Media Thickness	-0.552 (0.234)*
COVID-19/blood	-0.552 (0.266)*
Renal Insufficiency, Chronic/blood	-0.551 (0.214)*
Intensive Care Units, Pediatric/statistics & numerical data	-0.549 (0.251)*
Polymorphism, Genetic	-0.542 (0.245)*
Hypertension	-0.542 (0.257)*
Creatinine	-0.540 (0.246)*
Retinal Pigment Epithelium/pathology	-0.538 (0.289)
Magnetic Resonance Imaging/methods	-0.537 (0.111)***
Reference Values	-0.536 (0.139)***
ST Elevation Myocardial Infarction/physiopathology	-0.535 (0.325)
Respiration, Artificial/statistics & numerical data	-0.533 (0.190)**
Organ Size	-0.529 (0.183)**
Heart Conduction System/physiopathology	-0.526 (0.286)
Muscle, Skeletal/diagnostic imaging	-0.524 (0.283)
Predictive Value of Tests	-0.519 (0.048)***
Kidney Failure, Chronic/epidemiology	-0.512 (0.228)*
Heart Valve Prosthesis Implantation/instrumentation	-0.510 (0.352)

Atrial Fibrillation/etiology	-0.507 (0.270)
Fatigue/epidemiology	-0.503 (0.297)
Trauma Severity Indices	-0.501 (0.234)*
Athletes	-0.499 (0.192)**
Tomography, Optical Coherence/methods	-0.498 (0.108)***
Breast Neoplasms/genetics	-0.492 (0.266)
Risk	-0.492 (0.129)***
Acute Coronary Syndrome/complications	-0.488 (0.260)
Liver Cirrhosis/epidemiology	-0.484 (0.276)
Respiratory Distress Syndrome/mortality	-0.484 (0.301)
Noninvasive Ventilation	-0.482 (0.250)
Nomograms	-0.481 (0.216)*
Metabolic Syndrome/complications	-0.480 (0.288)
Creatinine/urine	-0.480 (0.295)
Crohn Disease/diagnosis	-0.477 (0.274)
Maternal Age	-0.477 (0.221)*
Renal Dialysis/mortality	-0.477 (0.259)
Pregnancy Trimester, Third	-0.475 (0.244)
Gait/physiology	-0.475 (0.254)
ROC Curve	-0.472 (0.077)***
Asthma/epidemiology	-0.471 (0.188)*
Survivors	-0.469 (0.202)*
Physical Functional Performance	-0.469 (0.273)
Electrocardiography/methods	-0.459 (0.226)*
Vitamin D Deficiency/complications	-0.456 (0.278)
Venous Thromboembolism/etiology	-0.449 (0.280)
Dyslipidemias/blood	-0.446 (0.323)
Ischemic Stroke	-0.444 (0.242)
Stroke/surgery	-0.441 (0.265)
Anthropometry	-0.440 (0.193)*
Cognitive Dysfunction/diagnosis	-0.437 (0.243)
Sleep Apnea, Obstructive/complications	-0.437 (0.248)
Shock, Septic/mortality	-0.436 (0.247)
Wounds and Injuries/complications	-0.435 (0.219)*
Melanoma/pathology	-0.433 (0.286)
Risk Assessment/statistics & numerical data	-0.432 (0.275)
Plaque, Atherosclerotic	-0.429 (0.257)
Immunohistochemistry	-0.427 (0.201)*
Acute Kidney Injury/chemically induced	-0.425 (0.266)
Visual Fields	-0.424 (0.269)
Peripheral Arterial Disease/epidemiology	-0.423 (0.317)
Hepatitis C, Chronic/complications	-0.422 (0.243)
Lung Neoplasms/mortality	-0.422 (0.303)
Brain Ischemia/physiopathology	-0.422 (0.317)
Parkinson Disease/complications	-0.420 (0.258)
Biomarkers/blood	-0.420 (0.054)***
Smartphone	-0.415 (0.240)
Prognosis	-0.415 (0.036)***
Social Class	-0.414 (0.178)*
Drug Prescriptions	-0.414 (0.223)
Serum Albumin/analysis	-0.414 (0.303)
Electroencephalography/methods	-0.413 (0.254)
Immunoglobulin G	-0.410 (0.240)
Optic Disk/pathology	-0.408 (0.251)
Healthcare Disparities/statistics & numerical data	-0.406 (0.252)

Patient Readmission/statistics & numerical data	-0.405 (0.134)**
Cardiovascular Diseases/physiopathology	-0.403 (0.236)
Ultrasonography, Prenatal/methods	-0.400 (0.220)
Frailty/diagnosis	-0.399 (0.236)
Substance-Related Disorders/epidemiology	-0.398 (0.219)
Fatigue/etiology	-0.398 (0.191)*
Neuropsychological Tests	-0.396 (0.130)**
Acute Kidney Injury/physiopathology	-0.395 (0.251)
Aortic Aneurysm, Abdominal/diagnostic imaging	-0.391 (0.303)
Peripheral Arterial Disease/mortality	-0.391 (0.294)
Biomarkers/metabolism	-0.387 (0.131)**
Angiogenesis Inhibitors/administration & dosage	-0.387 (0.254)
Diffusion Magnetic Resonance Imaging	-0.385 (0.280)
Neoplasms/diagnosis	-0.385 (0.233)
Aortic Valve/surgery	-0.383 (0.192)*
Atrial Fibrillation/therapy	-0.382 (0.199)
Ultrasonography, Prenatal	-0.382 (0.169)*
Attitude to Health	-0.381 (0.223)
Uterine Cervical Neoplasms/pathology	-0.376 (0.225)
Systole	-0.376 (0.263)
Diabetes Mellitus, Type 1/epidemiology	-0.375 (0.207)
Visual Fields/physiology	-0.373 (0.241)
Stroke	-0.372 (0.201)
Self Report	-0.372 (0.099)***
Eating	-0.372 (0.249)
Prevalence	-0.371 (0.043)***
Spectroscopy, Near-Infrared	-0.371 (0.208)
Brain/pathology	-0.365 (0.209)
Malnutrition/complications	-0.362 (0.272)
Diabetes Mellitus/blood	-0.361 (0.235)
Viral Load	-0.360 (0.146)*
Models, Statistical	-0.359 (0.220)
Healthcare Disparities	-0.358 (0.224)
Walk Test	-0.358 (0.256)
Inflammatory Bowel Diseases/complications	-0.356 (0.244)
Tomography, Optical Coherence	-0.356 (0.102)***
Stroke/diagnosis	-0.353 (0.143)*
Stroke Volume	-0.351 (0.131)**
Myocardial Infarction/complications	-0.351 (0.207)
Internationality	-0.351 (0.242)
Health Care Surveys	-0.351 (0.225)
Glomerular Filtration Rate	-0.351 (0.110)**
Kidney Failure, Chronic/mortality	-0.348 (0.217)
Drug Monitoring/methods	-0.343 (0.198)
Pain/epidemiology	-0.342 (0.256)
Risk Factors	-0.341 (0.026)***
Pain, Postoperative/etiology	-0.339 (0.184)
Cross-Sectional Studies	-0.339 (0.028)***
Diastole	-0.336 (0.296)
Sleep Apnea, Obstructive/epidemiology	-0.336 (0.278)
Echocardiography/methods	-0.336 (0.170)*
Immunoglobulin G/blood	-0.334 (0.216)
Ultrasonography/methods	-0.334 (0.129)**
Percutaneous Coronary Intervention/adverse effects	-0.331 (0.130)*
Sepsis/blood	-0.331 (0.204)

Biomarkers, Tumor/analysis	-0.330 (0.267)
Electroencephalography	-0.330 (0.172)
Preoperative Period	-0.328 (0.139)*
Venous Thromboembolism/epidemiology	-0.328 (0.253)
Hospital Mortality/trends	-0.327 (0.131)*
Pulmonary Veins/surgery	-0.327 (0.208)
Heart Failure/physiopathology	-0.325 (0.135)*
Anesthesia, General/adverse effects	-0.323 (0.203)
Vitamin D Deficiency/epidemiology	-0.321 (0.263)
Postoperative Complications/diagnosis	-0.321 (0.128)*
Sarcopenia/diagnosis	-0.321 (0.318)
Infant, Extremely Premature	-0.321 (0.212)
Delirium/diagnosis	-0.320 (0.248)
Up-Regulation	-0.320 (0.189)
Percutaneous Coronary Intervention	-0.319 (0.134)*
Assessment of Medication Adherence	-0.319 (0.147)*
Blood Glucose	-0.317 (0.152)*
Cardiovascular Diseases/diagnosis	-0.316 (0.159)*
Coronary Artery Bypass/adverse effects	-0.316 (0.190)
Adiposity	-0.316 (0.214)
Breast Neoplasms/epidemiology	-0.314 (0.214)
Bacteremia/mortality	-0.313 (0.286)
Heart Transplantation	-0.313 (0.262)
Positron-Emission Tomography	-0.312 (0.262)
Delivery, Obstetric/methods	-0.311 (0.234)
Angiotensin-Converting Enzyme Inhibitors/therapeutic use	-0.309 (0.241)
Electromyography	-0.307 (0.204)
RNA, Ribosomal, 16S/genetics	-0.307 (0.229)
Circadian Rhythm	-0.307 (0.260)
Diabetes Mellitus, Type 1/blood	-0.306 (0.193)
Medication Adherence	-0.306 (0.119)*
Gastrointestinal Microbiome	-0.305 (0.228)
Hemorrhage/therapy	-0.304 (0.259)
Neoplasm Grading	-0.304 (0.181)
Biomarkers/urine	-0.303 (0.189)
Lung/diagnostic imaging	-0.301 (0.148)*
Accelerometry	-0.301 (0.239)
Cesarean Section/statistics & numerical data	-0.301 (0.174)
Posture	-0.299 (0.226)
Age of Onset	-0.299 (0.154)
Asthma/drug therapy	-0.299 (0.196)
White People/statistics & numerical data	-0.296 (0.325)
Spinal Fusion/adverse effects	-0.294 (0.261)
Mental Status and Dementia Tests	-0.294 (0.286)
Primary Health Care/statistics & numerical data	-0.293 (0.184)
Critical Care	-0.292 (0.112)**
Cornea/pathology	-0.292 (0.213)
Lung/pathology	-0.291 (0.250)
Delirium/etiology	-0.291 (0.235)
Fractures, Bone/epidemiology	-0.291 (0.233)
Oximetry	-0.290 (0.207)
Brain Ischemia/etiology	-0.290 (0.274)
Immunocompromised Host	-0.287 (0.213)
Lymphatic Metastasis	-0.287 (0.194)
Multiple Sclerosis/complications	-0.286 (0.242)

Colorectal Neoplasms/drug therapy	-0.284 (0.227)
Vascular Stiffness	-0.284 (0.271)
Cross Infection/microbiology	-0.283 (0.226)
Glucose Tolerance Test	-0.283 (0.206)
Patient Compliance/statistics & numerical data	-0.283 (0.221)
Cardiopulmonary Resuscitation	-0.282 (0.178)
Patient Readmission	-0.281 (0.141)*
Liver Transplantation	-0.280 (0.181)
Brain Ischemia/epidemiology	-0.279 (0.258)
Feeding Behavior	-0.279 (0.169)
Case-Control Studies	-0.278 (0.043)***
Hip Fractures/epidemiology	-0.277 (0.250)
Hospital Mortality	-0.276 (0.057)***
Kidney/pathology	-0.276 (0.213)
Reoperation/statistics & numerical data	-0.275 (0.168)
Ontario/epidemiology	-0.274 (0.247)
Transplant Recipients	-0.273 (0.185)
Troponin T/blood	-0.272 (0.240)
Incidence	-0.271 (0.038)***
ST Elevation Myocardial Infarction/diagnosis	-0.270 (0.240)
Lactic Acid/blood	-0.270 (0.211)
Inpatients/statistics & numerical data	-0.270 (0.226)
Cognition/physiology	-0.269 (0.197)
C-Reactive Protein	-0.269 (0.255)
Hospitalization	-0.269 (0.057)***
C-Reactive Protein/metabolism	-0.269 (0.135)*
Early Detection of Cancer	-0.268 (0.219)
Hand Strength	-0.268 (0.171)
Postpartum Period	-0.267 (0.170)
Functional Status	-0.266 (0.250)
Arthroplasty, Replacement, Knee	-0.265 (0.246)
Stroke Volume/physiology	-0.265 (0.163)
Heart Defects, Congenital/surgery	-0.263 (0.176)
Anticoagulants/adverse effects	-0.262 (0.144)
Analgesics/therapeutic use	-0.262 (0.195)
Heart Rate/physiology	-0.261 (0.134)
Abdomen/surgery	-0.259 (0.245)
Weight Gain	-0.259 (0.176)
Antipsychotic Agents/therapeutic use	-0.258 (0.201)
Aortic Valve Stenosis/diagnostic imaging	-0.258 (0.280)
Premature Birth/epidemiology	-0.257 (0.178)
Black or African American	-0.257 (0.223)
Thrombosis/etiology	-0.256 (0.192)
Stroke/diagnostic imaging	-0.254 (0.210)
Heart Disease Risk Factors	-0.254 (0.172)
Diabetes Mellitus, Type 2	-0.253 (0.219)
Arthritis, Rheumatoid/epidemiology	-0.253 (0.212)
Choroid/pathology	-0.253 (0.205)
Diabetes Mellitus, Type 2/epidemiology	-0.252 (0.106)*
Osteoporotic Fractures/epidemiology	-0.252 (0.266)
Leukocyte Count	-0.251 (0.173)
Veterans	-0.250 (0.223)
Myocardial Infarction/etiology	-0.250 (0.185)
Cardiac Surgical Procedures/mortality	-0.247 (0.244)
Stomach Neoplasms/pathology	-0.246 (0.231)

Patient Discharge/statistics & numerical data	-0.246 (0.164)
Cardiac Output	-0.245 (0.243)
Postoperative Complications/physiopathology	-0.244 (0.183)
Ultrasonography	-0.243 (0.078)**
Macular Edema/diagnosis	-0.242 (0.331)
Troponin I/blood	-0.242 (0.268)
Visual Field Tests	-0.241 (0.258)
Magnetic Resonance Imaging	-0.241 (0.079)**
Inflammation/diagnosis	-0.240 (0.277)
Pulmonary Disease, Chronic Obstructive/epidemiology	-0.240 (0.168)
Medicare	-0.238 (0.154)
Lung Neoplasms/surgery	-0.238 (0.201)
Heart Valve Prosthesis Implantation/adverse effects	-0.237 (0.242)
Stroke/blood	-0.236 (0.267)
Emotions	-0.234 (0.183)
ST Elevation Myocardial Infarction/diagnostic imaging	-0.233 (0.279)
Sepsis	-0.233 (0.230)
Rural Population/statistics & numerical data	-0.233 (0.235)
Critical Illness/epidemiology	-0.233 (0.250)
Infant, Very Low Birth Weight	-0.232 (0.194)
Acute Kidney Injury/mortality	-0.232 (0.196)
Diabetes Mellitus, Type 1/complications	-0.231 (0.175)
Anti-Retroviral Agents/therapeutic use	-0.231 (0.204)
Postoperative Care	-0.231 (0.228)
Gastrectomy/methods	-0.230 (0.213)
Macular Edema/etiology	-0.230 (0.323)
Uric Acid/blood	-0.230 (0.234)
Neoplasms/psychology	-0.230 (0.223)
Myocardial Infarction/mortality	-0.229 (0.150)
Asthma/diagnosis	-0.228 (0.217)
Inflammation	-0.227 (0.176)
Stroke/complications	-0.224 (0.133)
Hypertension/diagnosis	-0.224 (0.177)
Macula Lutea/pathology	-0.224 (0.198)
Gestational Age	-0.224 (0.084)**
COVID-19/diagnosis	-0.223 (0.140)
Liver/pathology	-0.223 (0.194)
Tomography, X-Ray Computed	-0.223 (0.079)**
Recovery of Function/physiology	-0.223 (0.233)
Cardiac Surgical Procedures/adverse effects	-0.223 (0.125)
Autoantibodies/blood	-0.222 (0.221)
Pregnant People	-0.221 (0.227)
Urban Population	-0.221 (0.217)
Proportional Hazards Models	-0.220 (0.063)***
Peptide Fragments/blood	-0.220 (0.231)
Cardiovascular Diseases/etiology	-0.220 (0.147)
Cognition	-0.219 (0.119)
Cardiac Surgical Procedures	-0.219 (0.157)
Peripheral Arterial Disease/diagnosis	-0.219 (0.266)
Monitoring, Physiologic/methods	-0.219 (0.164)
Renal Dialysis	-0.218 (0.113)
Bilirubin/blood	-0.218 (0.252)
Morbidity	-0.217 (0.172)
Alcohol Drinking/epidemiology	-0.216 (0.221)
Cholesterol, HDL/blood	-0.215 (0.213)

Blood Glucose Self-Monitoring	-0.214 (0.216)
Biomarkers, Tumor/genetics	-0.214 (0.195)
Diabetes Mellitus, Type 2/complications	-0.213 (0.092)*
Dyslipidemias/epidemiology	-0.211 (0.252)
Independent Living	-0.210 (0.185)
Intensive Care Units	-0.210 (0.061)***
Fibrinolytic Agents/therapeutic use	-0.210 (0.195)
Practice Patterns, Physicians'/trends	-0.209 (0.196)
Geriatric Assessment/methods	-0.209 (0.162)
Depression/epidemiology	-0.206 (0.124)
Extracorporeal Membrane Oxygenation/adverse effects	-0.206 (0.223)
Stroke/etiology	-0.206 (0.132)
Inpatients	-0.205 (0.111)
Angiogenesis Inhibitors/therapeutic use	-0.204 (0.206)
C-Reactive Protein/analysis	-0.203 (0.135)
Epilepsy/drug therapy	-0.203 (0.202)
Practice Patterns, Physicians'/statistics & numerical data	-0.203 (0.138)
Anticoagulants/therapeutic use	-0.203 (0.119)
Computed Tomography Angiography	-0.202 (0.160)
Antirheumatic Agents/therapeutic use	-0.202 (0.136)
Mortality/trends	-0.202 (0.166)
Microcirculation	-0.201 (0.234)
Coronary Artery Bypass/methods	-0.201 (0.230)
Adrenal Cortex Hormones/administration & dosage	-0.200 (0.212)
Sepsis/diagnosis	-0.199 (0.168)
Postoperative Complications/diagnostic imaging	-0.199 (0.218)
Bone Density	-0.199 (0.169)
Emergency Service, Hospital/statistics & numerical data	-0.199 (0.089)*
Tumor Burden	-0.198 (0.202)
Hypertension/complications	-0.196 (0.143)
Diabetes Mellitus	-0.196 (0.167)
Injury Severity Score	-0.196 (0.131)
Extracorporeal Membrane Oxygenation	-0.194 (0.220)
Out-of-Hospital Cardiac Arrest/therapy	-0.194 (0.158)
Clopidogrel	-0.193 (0.269)
Cardiac Catheterization	-0.192 (0.219)
Kidney	-0.192 (0.218)
Platelet Aggregation Inhibitors/administration & dosage	-0.191 (0.208)
Sex Factors	-0.191 (0.062)**
Disability Evaluation	-0.191 (0.095)*
White People	-0.190 (0.194)
Gastrointestinal Agents/therapeutic use	-0.189 (0.212)
Heart Failure/mortality	-0.189 (0.132)
Logistic Models	-0.189 (0.054)***
Flow Cytometry	-0.189 (0.226)
Practice Patterns, Physicians'	-0.189 (0.148)
Health Behavior	-0.187 (0.171)
Out-of-Hospital Cardiac Arrest/mortality	-0.187 (0.182)
Health Resources/statistics & numerical data	-0.186 (0.231)
Endovascular Procedures	-0.186 (0.215)
Registries	-0.186 (0.043)***
Drug Prescriptions/statistics & numerical data	-0.185 (0.192)
Hospital Costs	-0.184 (0.199)
Health Status	-0.184 (0.096)
Malnutrition/diagnosis	-0.184 (0.243)

Time-to-Treatment/statistics & numerical data	-0.183 (0.196)
Blood Vessel Prosthesis Implantation/instrumentation	-0.183 (0.394)
Patient Admission	-0.182 (0.150)
Women's Health	-0.181 (0.268)
Prenatal Care	-0.181 (0.224)
California	-0.180 (0.210)
Absorptiometry, Photon	-0.179 (0.178)
Emergency Medical Services	-0.178 (0.124)
COVID-19/complications	-0.178 (0.096)
Blood Vessel Prosthesis Implantation/mortality	-0.178 (0.337)
Carcinoma, Squamous Cell/pathology	-0.177 (0.189)
Sepsis/therapy	-0.177 (0.225)
Colitis, Ulcerative/diagnosis	-0.175 (0.256)
Phacoemulsification	-0.175 (0.262)
Sleep Apnea, Obstructive/diagnosis	-0.175 (0.243)
Antibodies, Viral	-0.174 (0.212)
Defibrillators, Implantable	-0.174 (0.203)
Aortic Dissection/surgery	-0.173 (0.265)
Biomarkers/analysis	-0.173 (0.140)
Nutrition Assessment	-0.173 (0.169)
Blood Glucose/analysis	-0.172 (0.119)
Overweight/complications	-0.171 (0.248)
Ventricular Function, Right	-0.171 (0.268)
Spinal Cord Injuries/complications	-0.170 (0.240)
Pain, Postoperative/diagnosis	-0.169 (0.241)
Electric Impedance	-0.169 (0.179)
Critical Illness/mortality	-0.168 (0.162)
Pre-Eclampsia/diagnosis	-0.168 (0.286)
Neoplasms/epidemiology	-0.167 (0.151)
COVID-19/immunology	-0.167 (0.287)
Obesity/epidemiology	-0.167 (0.126)
Spirometry	-0.166 (0.198)
Phenotype	-0.165 (0.107)
Sex Distribution	-0.165 (0.161)
Treatment Failure	-0.165 (0.114)
Depression/diagnosis	-0.164 (0.190)
Antitubercular Agents/therapeutic use	-0.163 (0.195)
Analgesics, Opioid/adverse effects	-0.163 (0.173)
Percutaneous Coronary Intervention/mortality	-0.163 (0.167)
Patient Acuity	-0.162 (0.225)
Peritoneal Dialysis/adverse effects	-0.162 (0.245)
Oxygen Consumption/physiology	-0.162 (0.228)
Coronary Angiography	-0.161 (0.111)
Longitudinal Studies	-0.161 (0.040)***
Cohort Studies	-0.161 (0.028)***
Retina/pathology	-0.160 (0.200)
Acute Coronary Syndrome/diagnostic imaging	-0.160 (0.256)
Overweight/epidemiology	-0.159 (0.207)
Child Development	-0.158 (0.185)
Monitoring, Physiologic	-0.157 (0.211)
Disease Progression	-0.157 (0.054)**
Postoperative Complications/epidemiology	-0.156 (0.063)*
Biomechanical Phenomena	-0.154 (0.149)
Emergency Medical Services/methods	-0.154 (0.167)
Health Care Costs	-0.154 (0.135)

Mental Disorders/therapy	-0.153 (0.223)
Metformin/therapeutic use	-0.153 (0.213)
Mental Health	-0.151 (0.130)
Lymphocyte Count	-0.150 (0.222)
Machine Learning	-0.150 (0.157)
Myopia/physiopathology	-0.149 (0.280)
Body Temperature	-0.149 (0.215)
Multidetector Computed Tomography	-0.149 (0.248)
Cerebrovascular Circulation	-0.148 (0.185)
Transcatheter Aortic Valve Replacement/adverse effects	-0.147 (0.231)
Students	-0.147 (0.220)
Serum Albumin/metabolism	-0.146 (0.293)
COVID-19/mortality	-0.146 (0.142)
Arthroplasty, Replacement, Knee/adverse effects	-0.145 (0.196)
Liver Transplantation/adverse effects	-0.144 (0.185)
Heart Failure/diagnostic imaging	-0.144 (0.226)
Hypoglycemic Agents/administration & dosage	-0.144 (0.189)
Respiration, Artificial	-0.144 (0.095)
Apgar Score	-0.144 (0.243)
Patient Acceptance of Health Care	-0.144 (0.160)
Metabolic Syndrome/epidemiology	-0.143 (0.218)
Fibrinolytic Agents/administration & dosage	-0.142 (0.241)
Kidney Failure, Chronic/blood	-0.141 (0.234)
Prosthesis Failure	-0.141 (0.189)
Aortic Valve Stenosis/physiopathology	-0.141 (0.307)
Insulin Resistance	-0.141 (0.151)
Thrombolytic Therapy	-0.140 (0.228)
Cerebrovascular Circulation/physiology	-0.139 (0.182)
Microbial Sensitivity Tests	-0.139 (0.148)
Brain Injuries, Traumatic/complications	-0.139 (0.198)
Neoplasms/mortality	-0.138 (0.200)
Pregnancy Outcome/epidemiology	-0.138 (0.153)
Pulmonary Disease, Chronic Obstructive/physiopathology	-0.138 (0.157)
Critical Illness	-0.138 (0.081)
Hypoglycemia/chemically induced	-0.138 (0.220)
Sleep Apnea, Obstructive/physiopathology	-0.137 (0.268)
Cognitive Dysfunction/etiology	-0.137 (0.209)
Thromboembolism/prevention & control	-0.136 (0.245)
Pulmonary Disease, Chronic Obstructive/complications	-0.136 (0.182)
Workforce	-0.135 (0.213)
Lung Neoplasms/diagnosis	-0.135 (0.240)
Severity of Illness Index	-0.134 (0.038)***
Retina/diagnostic imaging	-0.134 (0.249)
Laparoscopy/adverse effects	-0.133 (0.161)
Linear Models	-0.133 (0.087)
Antineoplastic Agents/therapeutic use	-0.132 (0.126)
Heart Ventricles/diagnostic imaging	-0.132 (0.210)
Blood Pressure	-0.132 (0.099)
Patient Discharge	-0.130 (0.097)
Anxiety	-0.130 (0.196)
Infant, Low Birth Weight	-0.129 (0.216)
Birth Weight	-0.129 (0.128)
Heart Valve Prosthesis Implantation/methods	-0.128 (0.204)
Lung Neoplasms/therapy	-0.127 (0.225)
Platelet Aggregation Inhibitors/therapeutic use	-0.126 (0.155)

Fluorescein Angiography	-0.126 (0.162)
HIV Infections/complications	-0.126 (0.121)
Infant Mortality	-0.124 (0.243)
Aortic Valve Stenosis/surgery	-0.124 (0.222)
Biopsy	-0.124 (0.114)
Glomerular Filtration Rate/physiology	-0.124 (0.209)
Coronary Artery Disease/epidemiology	-0.123 (0.168)
Kidney Failure, Chronic/diagnosis	-0.121 (0.234)
Physicians	-0.121 (0.159)
ErbB Receptors/genetics	-0.121 (0.295)
Hospitalization/statistics & numerical data	-0.121 (0.070)
Colorectal Neoplasms/diagnosis	-0.121 (0.204)
Oximetry/methods	-0.120 (0.239)
Hemorrhage/mortality	-0.120 (0.268)
Peripheral Arterial Disease/physiopathology	-0.120 (0.277)
Kidney Transplantation/adverse effects	-0.120 (0.144)
Carcinoma, Hepatocellular/pathology	-0.119 (0.374)
Intensive Care Units, Pediatric	-0.118 (0.145)
Sedentary Behavior	-0.118 (0.172)
Tomography, X-Ray Computed/methods	-0.118 (0.122)
Walking	-0.118 (0.168)
Health Surveys	-0.117 (0.186)
Military Personnel	-0.117 (0.217)
Neoplasm Metastasis	-0.116 (0.184)
Atrial Fibrillation/surgery	-0.116 (0.175)
Knee Joint/surgery	-0.116 (0.236)
Betacoronavirus	-0.116 (0.221)
Parents	-0.115 (0.177)
Psychiatric Status Rating Scales	-0.115 (0.142)
Coronary Artery Bypass	-0.115 (0.213)
Endovascular Procedures/adverse effects	-0.115 (0.177)
Cholesterol/blood	-0.115 (0.212)
Chronic Pain/epidemiology	-0.115 (0.244)
Fluid Therapy/methods	-0.114 (0.196)
Atrial Fibrillation/drug therapy	-0.114 (0.149)
Polypharmacy	-0.114 (0.177)
Neutrophils	-0.113 (0.299)
Blood Gas Analysis	-0.113 (0.208)
Lung Neoplasms/drug therapy	-0.111 (0.260)
Databases, Factual	-0.110 (0.068)
Mortality	-0.109 (0.140)
Liver Neoplasms/secondary	-0.108 (0.242)
Cross Infection/epidemiology	-0.108 (0.178)
HIV Infections/epidemiology	-0.107 (0.124)
Pulmonary Disease, Chronic Obstructive/diagnosis	-0.106 (0.171)
Respiration, Artificial/adverse effects	-0.106 (0.172)
Nursing Homes	-0.104 (0.190)
Walking/physiology	-0.104 (0.204)
Blood Pressure/physiology	-0.104 (0.122)
Diabetes Mellitus/epidemiology	-0.103 (0.122)
Colorectal Neoplasms/surgery	-0.103 (0.165)
Stroke/epidemiology	-0.103 (0.115)
Heart-Assist Devices	-0.101 (0.237)
Adalimumab/therapeutic use	-0.101 (0.205)
Regression Analysis	-0.101 (0.097)

Spinal Fusion/methods	-0.100 (0.210)
Mitral Valve/diagnostic imaging	-0.099 (0.279)
Adolescent	-0.098 (0.031)**
Mothers	-0.098 (0.187)
Mutation	-0.097 (0.122)
Primary Health Care	-0.097 (0.106)
Organ Dysfunction Scores	-0.096 (0.170)
Critical Care/methods	-0.095 (0.148)
Insulin/therapeutic use	-0.094 (0.164)
Anesthesia, General	-0.094 (0.162)
Multivariate Analysis	-0.093 (0.060)
Hand Strength/physiology	-0.093 (0.249)
Motivation	-0.093 (0.185)
Sepsis/drug therapy	-0.093 (0.200)
Sleep	-0.093 (0.138)
Emergency Medical Services/statistics & numerical data	-0.092 (0.178)
Drug-Eluting Stents	-0.092 (0.174)
Myocardial Infarction/epidemiology	-0.092 (0.143)
Quality Indicators, Health Care	-0.091 (0.192)
Echocardiography	-0.091 (0.096)
Postoperative Complications/etiology	-0.091 (0.070)
Surgical Wound Infection/epidemiology	-0.090 (0.193)
Proton Pump Inhibitors/therapeutic use	-0.090 (0.221)
Motor Activity	-0.089 (0.217)
Heart Valve Prosthesis Implantation/mortality	-0.089 (0.256)
SARS-CoV-2	-0.089 (0.068)
Patient Reported Outcome Measures	-0.089 (0.097)
Electrocardiography	-0.089 (0.101)
Osteoarthritis, Knee/surgery	-0.088 (0.211)
Tumor Necrosis Factor-alpha/antagonists & inhibitors	-0.088 (0.155)
Liver Cirrhosis/complications	-0.088 (0.160)
Muscle Strength	-0.087 (0.201)
Acute Coronary Syndrome/epidemiology	-0.086 (0.228)
Arrhythmias, Cardiac/physiopathology	-0.085 (0.306)
Heart Failure/blood	-0.085 (0.210)
Analysis of Variance	-0.085 (0.137)
Sarcopenia/epidemiology	-0.084 (0.272)
Deglutition Disorders/etiology	-0.084 (0.227)
Inflammation/blood	-0.083 (0.198)
Hemorrhage/epidemiology	-0.083 (0.173)
Pneumonia/diagnosis	-0.082 (0.260)
Physician-Patient Relations	-0.082 (0.166)
Depression/etiology	-0.081 (0.183)
Depression/psychology	-0.081 (0.141)
Confidence Intervals	-0.081 (0.228)
Perception	-0.081 (0.169)
Glucose	-0.080 (0.217)
Cesarean Section/adverse effects	-0.079 (0.193)
Neoadjuvant Therapy	-0.078 (0.186)
Coronary Angiography/methods	-0.078 (0.177)
Fluorescein Angiography/methods	-0.078 (0.190)
Postoperative Period	-0.078 (0.087)
ST Elevation Myocardial Infarction/mortality	-0.077 (0.244)
Syndrome	-0.077 (0.173)
Echocardiography, Transesophageal	-0.075 (0.213)

Population Surveillance	-0.075 (0.197)
Platelet Count	-0.074 (0.170)
Cesarean Section	-0.073 (0.147)
Analgesics, Opioid/therapeutic use	-0.072 (0.124)
Heart Failure/epidemiology	-0.072 (0.133)
Waist Circumference	-0.072 (0.196)
SARS-CoV-2/isolation & purification	-0.072 (0.243)
Respiratory Function Tests	-0.070 (0.152)
Blood Flow Velocity/physiology	-0.069 (0.243)
Percutaneous Coronary Intervention/methods	-0.069 (0.161)
Aortic Valve Stenosis/diagnosis	-0.069 (0.268)
Ventricular Dysfunction, Left/physiopathology	-0.069 (0.227)
Aortic Aneurysm, Abdominal/surgery	-0.068 (0.294)
Exercise	-0.068 (0.097)
Antidepressive Agents/therapeutic use	-0.068 (0.195)
Oxygen	-0.066 (0.159)
Guideline Adherence/statistics & numerical data	-0.066 (0.170)
Anxiety/etiology	-0.065 (0.202)
Exercise Test/methods	-0.064 (0.247)
Fibrinolytic Agents/adverse effects	-0.064 (0.269)
Cluster Analysis	-0.063 (0.224)
Patient Satisfaction/statistics & numerical data	-0.062 (0.171)
Prostatic Neoplasms/surgery	-0.062 (0.226)
Hepatitis C/drug therapy	-0.062 (0.232)
Postmenopause	-0.062 (0.190)
Renal Insufficiency, Chronic/epidemiology	-0.062 (0.149)
Anesthetics, Local/administration & dosage	-0.061 (0.229)
Ethnicity	-0.060 (0.161)
Propensity Score	-0.060 (0.071)
Extracorporeal Membrane Oxygenation/methods	-0.060 (0.188)
Disease-Free Survival	-0.060 (0.095)
Subarachnoid Hemorrhage/complications	-0.059 (0.212)
Europe/epidemiology	-0.059 (0.139)
Cardiac Catheterization/adverse effects	-0.058 (0.232)
Heart Failure	-0.058 (0.200)
Kaplan-Meier Estimate	-0.058 (0.067)
Aging	-0.057 (0.187)
Pregnancy Outcome	-0.057 (0.111)
Aspirin/therapeutic use	-0.056 (0.216)
Odds Ratio	-0.056 (0.074)
Pancreatic Neoplasms/surgery	-0.056 (0.220)
Female	-0.056 (0.031)
Brain Ischemia/diagnostic imaging	-0.055 (0.289)
Exercise/physiology	-0.054 (0.133)
Carotid Artery Diseases/diagnostic imaging	-0.054 (0.314)
HIV Infections/immunology	-0.054 (0.208)
Crohn Disease/drug therapy	-0.054 (0.165)
Blood Glucose/metabolism	-0.054 (0.111)
Palliative Care/methods	-0.054 (0.194)
Parents/psychology	-0.053 (0.192)
Stress, Psychological/psychology	-0.052 (0.192)
Cardiopulmonary Resuscitation/methods	-0.052 (0.172)
Rectal Neoplasms/surgery	-0.052 (0.198)
Skin Neoplasms/pathology	-0.051 (0.221)
Survival Analysis	-0.051 (0.078)

Bone Density/physiology	-0.051 (0.225)
Lung	-0.048 (0.172)
Surveys and Questionnaires	-0.046 (0.038)
Seasons	-0.046 (0.136)
Antihypertensive Agents/therapeutic use	-0.046 (0.152)
Cardiovascular Diseases/epidemiology	-0.046 (0.106)
Polysomnography	-0.046 (0.175)
Coronary Circulation	-0.046 (0.257)
Pregnancy Complications, Infectious/epidemiology	-0.045 (0.221)
Comorbidity	-0.045 (0.056)
Pelvic Organ Prolapse/surgery	-0.044 (0.226)
Acute Disease	-0.044 (0.078)
Infant, Small for Gestational Age	-0.043 (0.196)
Triglycerides/blood	-0.043 (0.173)
Atrial Fibrillation/epidemiology	-0.043 (0.138)
Activities of Daily Living	-0.042 (0.094)
Diabetic Nephropathies/diagnosis	-0.042 (0.293)
Cardiovascular Diseases/mortality	-0.042 (0.145)
Body Mass Index	-0.042 (0.058)
Non-alcoholic Fatty Liver Disease/epidemiology	-0.041 (0.272)
Lung/physiopathology	-0.040 (0.145)
Aged	-0.039 (0.023)
Anti-Bacterial Agents/adverse effects	-0.039 (0.165)
Kidney Failure, Chronic/complications	-0.038 (0.185)
Age Factors	-0.037 (0.049)
Hypothermia, Induced	-0.037 (0.245)
Cardiovascular Agents/therapeutic use	-0.037 (0.240)
Coronary Artery Disease/physiopathology	-0.036 (0.183)
Sepsis/mortality	-0.036 (0.164)
Renal Insufficiency, Chronic/physiopathology	-0.035 (0.177)
Amputation, Surgical	-0.035 (0.243)
Shock, Cardiogenic/therapy	-0.035 (0.311)
Acute Kidney Injury/etiology	-0.032 (0.149)
Cervical Vertebrae/surgery	-0.031 (0.224)
Heart Failure/diagnosis	-0.030 (0.131)
Brain/diagnostic imaging	-0.030 (0.151)
Diabetes, Gestational/diagnosis	-0.030 (0.276)
Microcirculation/physiology	-0.029 (0.236)
Radiography	-0.029 (0.126)
Heart Failure/etiology	-0.029 (0.211)
Cost of Illness	-0.028 (0.117)
Arterial Pressure	-0.028 (0.210)
Coronary Artery Disease/complications	-0.027 (0.180)
Renal Insufficiency, Chronic/mortality	-0.027 (0.220)
Non-ST Elevated Myocardial Infarction/mortality	-0.026 (0.290)
Glycated Hemoglobin/metabolism	-0.025 (0.135)
Nutritional Status	-0.024 (0.110)
Infant	-0.023 (0.046)
Acute Kidney Injury/epidemiology	-0.022 (0.159)
Pain Measurement/methods	-0.022 (0.153)
Patient Selection	-0.022 (0.119)
Retrospective Studies	-0.022 (0.020)
Cystic Fibrosis/complications	-0.021 (0.223)
Diabetes Mellitus, Type 2/metabolism	-0.021 (0.229)
Intensive Care Units/statistics & numerical data	-0.020 (0.107)

Aging/physiology	-0.020 (0.205)
Male	-0.020 (0.030)
Ontario	-0.020 (0.213)
COVID-19/psychology	-0.019 (0.205)
Prostatic Neoplasms/pathology	-0.019 (0.197)
Perioperative Care/methods	-0.019 (0.217)
Antirheumatic Agents/administration & dosage	-0.017 (0.232)
Thrombolytic Therapy/adverse effects	-0.017 (0.294)
Hospitalization/trends	-0.016 (0.158)
Diabetic Retinopathy/diagnosis	-0.015 (0.216)
Polymerase Chain Reaction	-0.015 (0.202)
Aged, 80 and over	-0.014 (0.025)
Length of Stay/trends	-0.013 (0.217)
Bacteria/isolation & purification	-0.013 (0.211)
Feces/microbiology	-0.013 (0.218)
Postural Balance/physiology	-0.012 (0.206)
Coronary Artery Disease/diagnosis	-0.011 (0.156)
Fundus Oculi	-0.010 (0.177)
Arthritis, Psoriatic/drug therapy	-0.010 (0.208)
Pregnancy	-0.010 (0.053)
Urinary Tract Infections/epidemiology	-0.009 (0.227)
Diabetes Mellitus, Type 2/mortality	-0.007 (0.203)
Respiration, Artificial/methods	-0.007 (0.154)
Acute Coronary Syndrome/mortality	-0.006 (0.209)
APACHE	-0.006 (0.148)
Glaucoma/physiopathology	-0.006 (0.251)
Forecasting	-0.006 (0.199)
Catheter Ablation	-0.006 (0.223)
Child, Preschool	-0.005 (0.048)
Enteral Nutrition	-0.005 (0.226)
Blood Transfusion	-0.004 (0.162)
Socioeconomic Factors	-0.004 (0.081)
Ventricular Function, Left/physiology	-0.003 (0.184)
Heart Atria/diagnostic imaging	-0.003 (0.286)
ST Elevation Myocardial Infarction/surgery	-0.003 (0.270)
Reproducibility of Results	-0.003 (0.062)
Anti-Inflammatory Agents/therapeutic use	-0.003 (0.195)
Heart Failure/complications	-0.002 (0.146)
Stroke/drug therapy	-0.002 (0.205)
Trauma Centers	-0.002 (0.157)
Age Distribution	-0.002 (0.153)
Rectum/surgery	-0.001 (0.228)
Atrial Fibrillation/mortality	-0.001 (0.188)
Diabetes Mellitus, Type 2/physiopathology	-0.001 (0.179)
Delivery of Health Care	-0.001 (0.150)
COVID-19/therapy	0.000 (0.121)
Renal Replacement Therapy	0.001 (0.250)
Emergency Service, Hospital	0.002 (0.066)
Acute Kidney Injury/diagnosis	0.002 (0.161)
Risk Assessment	0.003 (0.046)
Range of Motion, Articular/physiology	0.003 (0.195)
Percutaneous Coronary Intervention/instrumentation	0.004 (0.190)
Blood Flow Velocity	0.004 (0.181)
Malnutrition/epidemiology	0.004 (0.198)
Mobility Limitation	0.004 (0.241)

Costs and Cost Analysis	0.005 (0.207)
Postural Balance	0.005 (0.218)
Creatinine/blood	0.006 (0.137)
Quality of Health Care	0.007 (0.152)
Hepacivirus/genetics	0.007 (0.247)
Cognitive Dysfunction/epidemiology	0.007 (0.221)
Colorectal Neoplasms/mortality	0.008 (0.234)
Arthroplasty, Replacement, Hip/adverse effects	0.009 (0.188)
Diabetes Mellitus, Type 2/blood	0.010 (0.123)
Exercise Test	0.011 (0.145)
Oxygen/metabolism	0.011 (0.209)
Drug Monitoring	0.011 (0.207)
Pain	0.012 (0.134)
Sepsis/complications	0.012 (0.172)
Warfarin/therapeutic use	0.012 (0.277)
Heart Ventricles/physiopathology	0.013 (0.226)
Atrial Fibrillation/complications	0.013 (0.139)
Fibrosis	0.013 (0.201)
Parity	0.014 (0.182)
Brain Ischemia/diagnosis	0.014 (0.253)
Infliximab/therapeutic use	0.014 (0.197)
Angiotensin Receptor Antagonists/therapeutic use	0.014 (0.267)
Administration, Inhalation	0.015 (0.176)
Middle Aged	0.015 (0.024)
Intubation, Intratracheal/adverse effects	0.015 (0.195)
Hypertension/physiopathology	0.015 (0.167)
Blood Vessel Prosthesis Implantation/adverse effects	0.016 (0.262)
Atrial Fibrillation/diagnostic imaging	0.016 (0.243)
Patient Admission/statistics & numerical data	0.017 (0.161)
Critical Illness/therapy	0.017 (0.120)
Coronary Artery Disease/diagnostic imaging	0.018 (0.140)
Cause of Death	0.019 (0.101)
Adult	0.019 (0.020)
Coronary Vessels/pathology	0.019 (0.263)
Academic Medical Centers	0.020 (0.165)
Nerve Fibers/pathology	0.020 (0.293)
Hemorrhage/etiology	0.021 (0.181)
Time Factors	0.021 (0.031)
Chemotherapy, Adjuvant	0.023 (0.171)
Catheterization, Central Venous/adverse effects	0.023 (0.205)
Anticoagulants/administration & dosage	0.023 (0.146)
Neoplasm Staging	0.024 (0.086)
Hospitals, Pediatric	0.024 (0.199)
Kidney Transplantation	0.024 (0.150)
Regional Blood Flow	0.024 (0.219)
Chronic Pain/drug therapy	0.025 (0.229)
Prospective Studies	0.026 (0.019)
Myocardial Infarction/therapy	0.026 (0.167)
Ankle Brachial Index	0.026 (0.259)
Arthritis, Rheumatoid/drug therapy	0.026 (0.147)
Anxiety/psychology	0.026 (0.158)
Sepsis/epidemiology	0.026 (0.170)
Video Recording	0.026 (0.166)
Registries/statistics & numerical data	0.028 (0.160)
Retinal Vessels/diagnostic imaging	0.029 (0.205)

Inflammatory Bowel Diseases/drug therapy	0.029 (0.174)
Hydroxymethylglutaryl-CoA Reductase Inhibitors/therapeutic use	0.030 (0.137)
Breast Neoplasms/diagnostic imaging	0.030 (0.225)
Neoplasms/drug therapy	0.030 (0.135)
COVID-19/epidemiology	0.030 (0.071)
Colorectal Neoplasms/epidemiology	0.031 (0.242)
Health Expenditures	0.032 (0.223)
Elective Surgical Procedures/adverse effects	0.032 (0.208)
Glaucoma, Open-Angle/diagnosis	0.033 (0.267)
Renal Insufficiency, Chronic/therapy	0.034 (0.168)
Premature Birth	0.036 (0.232)
Sexual Behavior	0.036 (0.213)
Breast Neoplasms/therapy	0.036 (0.199)
Continuous Positive Airway Pressure	0.038 (0.249)
Biological Products/therapeutic use	0.038 (0.177)
Renal Dialysis/adverse effects	0.039 (0.146)
Shock, Septic/blood	0.039 (0.291)
COVID-19	0.039 (0.082)
Heart Rate	0.039 (0.127)
Anxiety/epidemiology	0.042 (0.158)
Peripheral Arterial Disease/therapy	0.042 (0.272)
Obesity, Morbid/surgery	0.042 (0.148)
Genotype	0.042 (0.113)
General Practice	0.044 (0.209)
Internet	0.044 (0.194)
Infant, Newborn	0.045 (0.046)
Frail Elderly	0.045 (0.173)
Thrombectomy/methods	0.045 (0.207)
Liver Cirrhosis/diagnosis	0.046 (0.234)
Pilot Projects	0.047 (0.059)
Diabetes Mellitus/mortality	0.047 (0.255)
Neoplasms/complications	0.047 (0.134)
Stroke/physiopathology	0.048 (0.163)
Hypoglycemia/epidemiology	0.050 (0.227)
Pregnancy Trimester, First	0.050 (0.185)
Genetic Predisposition to Disease	0.050 (0.149)
Electric Countershock/instrumentation	0.050 (0.289)
Oxygen Consumption	0.051 (0.201)
Stroke/therapy	0.051 (0.150)
Acute Coronary Syndrome/diagnosis	0.051 (0.184)
Chronic Disease	0.052 (0.073)
Survival Rate/trends	0.052 (0.106)
Ferritins/blood	0.054 (0.211)
Tidal Volume	0.056 (0.216)
Infant, Premature	0.058 (0.098)
Hospitals	0.058 (0.079)
Renal Insufficiency, Chronic/diagnosis	0.059 (0.176)
Oxidative Stress	0.059 (0.230)
Peripheral Arterial Disease/diagnostic imaging	0.060 (0.317)
Laparoscopy	0.060 (0.132)
Adrenal Cortex Hormones/therapeutic use	0.061 (0.141)
Postoperative Complications/mortality	0.062 (0.129)
Atherosclerosis/blood	0.063 (0.285)
Opioid-Related Disorders/drug therapy	0.063 (0.211)
Follow-Up Studies	0.063 (0.030)*

Accidental Falls	0.064 (0.244)
Obesity/diagnosis	0.064 (0.202)
Neoplasms/therapy	0.065 (0.156)
Delirium/epidemiology	0.065 (0.218)
Blood Transfusion/statistics & numerical data	0.066 (0.227)
Patient Education as Topic	0.066 (0.202)
Pain Measurement	0.069 (0.083)
Atrial Fibrillation/physiopathology	0.069 (0.161)
Liver Neoplasms/mortality	0.070 (0.222)
Drug-Related Side Effects and Adverse Reactions/epidemiology	0.070 (0.161)
Pressure	0.070 (0.167)
Catheter Ablation/adverse effects	0.071 (0.180)
Wounds and Injuries/therapy	0.071 (0.182)
Death, Sudden, Cardiac/epidemiology	0.072 (0.280)
Child	0.072 (0.037)*
Renal Insufficiency, Chronic/complications	0.072 (0.133)
East Asian People	0.072 (0.164)
Young Adult	0.073 (0.029)*
Randomized Controlled Trials as Topic	0.074 (0.136)
Survival Rate	0.074 (0.071)
Intubation, Intratracheal	0.075 (0.176)
Atrial Fibrillation/blood	0.075 (0.276)
Tertiary Care Centers	0.075 (0.071)
Interviews as Topic	0.076 (0.218)
Emergencies	0.076 (0.177)
Coronary Artery Disease/blood	0.076 (0.190)
Coronary Artery Disease/surgery	0.076 (0.151)
Patient Compliance	0.076 (0.145)
Health Status Indicators	0.076 (0.213)
Health Care Costs/statistics & numerical data	0.077 (0.176)
COVID-19/physiopathology	0.077 (0.223)
Pregnancy Complications/epidemiology	0.077 (0.160)
Drug Resistance, Multiple, Bacterial	0.078 (0.196)
Neoplasm Recurrence, Local	0.079 (0.161)
Immunosuppressive Agents/adverse effects	0.080 (0.168)
Adrenergic beta-Antagonists/therapeutic use	0.080 (0.192)
Intensive Care Units, Neonatal	0.080 (0.132)
Neoplasm Recurrence, Local/pathology	0.081 (0.150)
Smoking/epidemiology	0.082 (0.148)
Anti-Bacterial Agents/therapeutic use	0.082 (0.070)
Catheterization, Peripheral/adverse effects	0.082 (0.192)
Educational Status	0.082 (0.142)
Parturition	0.083 (0.214)
Body Composition	0.083 (0.142)
Delivery, Obstetric	0.084 (0.191)
Carcinoma, Non-Small-Cell Lung/pathology	0.085 (0.257)
COVID-19 Drug Treatment	0.085 (0.156)
Frailty/epidemiology	0.086 (0.204)
Lower Extremity/blood supply	0.086 (0.213)
Anesthesia, General/methods	0.086 (0.191)
Feces/chemistry	0.086 (0.214)
Anti-Inflammatory Agents, Non-Steroidal/therapeutic use	0.087 (0.179)
Hospitalization/economics	0.087 (0.172)
Blood Vessel Prosthesis	0.088 (0.309)
Anesthesia	0.088 (0.219)

HIV Infections/drug therapy	0.088 (0.128)
Glaucoma, Open-Angle/physiopathology	0.088 (0.255)
Macular Edema/drug therapy	0.088 (0.286)
Atrial Fibrillation/diagnosis	0.089 (0.138)
Health Personnel	0.089 (0.141)
Postoperative Complications	0.089 (0.107)
Glycated Hemoglobin/analysis	0.090 (0.116)
Dyspnea/etiology	0.090 (0.223)
Muscle Strength/physiology	0.093 (0.173)
Carcinoma, Non-Small-Cell Lung/drug therapy	0.093 (0.306)
Peripheral Arterial Disease/surgery	0.095 (0.255)
Arthritis, Rheumatoid/diagnosis	0.095 (0.184)
Breast Neoplasms/mortality	0.096 (0.218)
Diabetic Retinopathy/complications	0.096 (0.297)
Risk Assessment/methods	0.096 (0.092)
Breast Neoplasms/surgery	0.096 (0.153)
Kidney/drug effects	0.096 (0.237)
Fluid Therapy	0.097 (0.226)
Length of Stay/statistics & numerical data	0.098 (0.076)
Recurrence	0.098 (0.062)
Antineoplastic Combined Chemotherapy Protocols/therapeutic use	0.098 (0.112)
Coronary Artery Disease/therapy	0.098 (0.158)
Diabetes Mellitus, Type 2/diagnosis	0.100 (0.140)
Obesity/complications	0.101 (0.108)
Influenza, Human/epidemiology	0.101 (0.192)
Diagnosis, Differential	0.101 (0.131)
Graft Survival	0.102 (0.160)
Nurses	0.102 (0.220)
Ventricular Function, Left	0.104 (0.142)
Body Weight	0.104 (0.122)
Humans	0.104 (0.177)
Causality	0.104 (0.230)
HIV Infections/virology	0.104 (0.186)
Practice Guidelines as Topic	0.105 (0.107)
Warfarin/adverse effects	0.105 (0.240)
Sensitivity and Specificity	0.106 (0.070)
Endovascular Procedures/mortality	0.106 (0.266)
Reoperation	0.106 (0.121)
Social Support	0.107 (0.161)
Thrombelastography	0.108 (0.240)
Secondary Prevention	0.110 (0.208)
Sleep/physiology	0.110 (0.171)
Arteriovenous Shunt, Surgical/adverse effects	0.111 (0.265)
Elective Surgical Procedures	0.112 (0.148)
Depression/complications	0.112 (0.221)
Diabetes, Gestational/epidemiology	0.113 (0.231)
Pancreatic Neoplasms/pathology	0.114 (0.224)
Breast Neoplasms/diagnosis	0.114 (0.218)
Visual Acuity	0.115 (0.087)
Tissue Donors	0.117 (0.182)
Blood Coagulation	0.117 (0.232)
Hypertension, Pulmonary/physiopathology	0.118 (0.232)
Osteoporosis/epidemiology	0.118 (0.256)
Neoplasm Recurrence, Local/epidemiology	0.118 (0.189)
Bariatric Surgery	0.119 (0.176)

Platelet Aggregation Inhibitors/adverse effects	0.119 (0.183)
Coronary Artery Disease/mortality	0.119 (0.154)
Liver/diagnostic imaging	0.120 (0.251)
Rural Population	0.121 (0.166)
Echocardiography, Doppler	0.121 (0.199)
Retinal Ganglion Cells/pathology	0.122 (0.296)
Europe	0.124 (0.116)
Early Diagnosis	0.124 (0.133)
Demography	0.125 (0.213)
Morbidity/trends	0.127 (0.239)
Anemia/epidemiology	0.128 (0.192)
Hispanic or Latino	0.129 (0.254)
Secondary Prevention/methods	0.131 (0.230)
Antihypertensive Agents/adverse effects	0.131 (0.243)
Heart Arrest/therapy	0.132 (0.181)
Lymph Nodes/pathology	0.132 (0.183)
Radiopharmaceuticals	0.132 (0.322)
Asian People	0.133 (0.147)
Pneumonia/epidemiology	0.133 (0.202)
Intraocular Pressure	0.133 (0.154)
Hypoglycemic Agents/therapeutic use	0.133 (0.128)
Chi-Square Distribution	0.134 (0.096)
Critical Care/statistics & numerical data	0.135 (0.216)
Anxiety/diagnosis	0.135 (0.251)
Obesity/surgery	0.135 (0.190)
Pacemaker, Artificial	0.135 (0.218)
Myocardial Infarction/blood	0.136 (0.222)
Neoplasm Invasiveness	0.136 (0.203)
Heart Failure/drug therapy	0.137 (0.158)
Postoperative Care/methods	0.137 (0.200)
Shock, Cardiogenic/mortality	0.138 (0.317)
Aortic Valve/diagnostic imaging	0.138 (0.271)
Hemodynamics	0.139 (0.108)
Patient Outcome Assessment	0.140 (0.191)
Cardiovascular Agents/adverse effects	0.140 (0.253)
Range of Motion, Articular	0.141 (0.150)
Surgical Wound Infection/etiology	0.141 (0.221)
Statistics, Nonparametric	0.141 (0.130)
Personal Satisfaction	0.141 (0.170)
Energy Intake	0.142 (0.157)
Ultrasonography, Doppler	0.142 (0.195)
Accidental Falls/statistics & numerical data	0.142 (0.187)
Length of Stay	0.142 (0.064)*
Asthma/physiopathology	0.143 (0.216)
Visual Acuity/physiology	0.143 (0.119)
Attitude of Health Personnel	0.143 (0.134)
Arthritis, Rheumatoid/complications	0.144 (0.200)
Universities	0.146 (0.201)
Analgesics, Opioid/administration & dosage	0.147 (0.158)
Blood Glucose/drug effects	0.148 (0.192)
Income	0.148 (0.227)
HIV Infections/diagnosis	0.148 (0.204)
Bacteremia/drug therapy	0.149 (0.226)
Diabetes Mellitus, Type 2/therapy	0.149 (0.178)
Hydroxymethylglutaryl-CoA Reductase Inhibitors/adverse effects	0.150 (0.227)

Diabetes Mellitus, Type 2/drug therapy	0.150 (0.120)
Single-Blind Method	0.151 (0.197)
Drug Interactions	0.152 (0.189)
Vitamin D/analogs & derivatives	0.153 (0.279)
Immunologic Factors/therapeutic use	0.153 (0.198)
Preoperative Care/methods	0.154 (0.157)
Antineoplastic Agents/adverse effects	0.154 (0.147)
COVID-19/virology	0.155 (0.239)
Adenocarcinoma/surgery	0.156 (0.232)
SARS-CoV-2/immunology	0.156 (0.290)
Aortic Aneurysm, Abdominal/mortality	0.156 (0.343)
Acute Coronary Syndrome/therapy	0.160 (0.200)
Asymptomatic Diseases	0.160 (0.200)
Kidney Failure, Chronic/therapy	0.160 (0.146)
Anti-Bacterial Agents/administration & dosage	0.161 (0.127)
Transcatheter Aortic Valve Replacement/instrumentation	0.161 (0.333)
Intubation, Intratracheal/methods	0.161 (0.161)
Endovascular Procedures/methods	0.162 (0.168)
Adaptation, Psychological	0.163 (0.163)
Protein Precursors/blood	0.163 (0.275)
Respiratory Tract Infections/epidemiology	0.163 (0.207)
Hemorrhage/chemically induced	0.165 (0.142)
Palliative Care	0.165 (0.163)
Antiviral Agents/administration & dosage	0.165 (0.229)
Antibodies, Monoclonal, Humanized/administration & dosage	0.166 (0.201)
Tonometry, Ocular	0.166 (0.202)
Aortic Valve/physiopathology	0.166 (0.334)
Drug Resistance, Bacterial	0.166 (0.193)
Contrast Media	0.167 (0.179)
Contrast Media/adverse effects	0.167 (0.251)
Antiviral Agents/therapeutic use	0.168 (0.123)
Brain Ischemia/drug therapy	0.168 (0.280)
Vital Capacity	0.169 (0.212)
Action Potentials	0.169 (0.237)
Delivery, Obstetric/statistics & numerical data	0.170 (0.215)
Ventricular Remodeling	0.171 (0.239)
Stress Disorders, Post-Traumatic/epidemiology	0.171 (0.235)
Protective Factors	0.172 (0.168)
Stroke/mortality	0.172 (0.149)
Antirheumatic Agents/adverse effects	0.173 (0.185)
Ischemic Stroke/diagnosis	0.173 (0.248)
Lumbar Vertebrae/surgery	0.177 (0.154)
Resuscitation/methods	0.178 (0.213)
Cognitive Dysfunction/psychology	0.179 (0.265)
Immunosuppressive Agents/therapeutic use	0.180 (0.112)
Antiviral Agents/adverse effects	0.180 (0.212)
Drug Costs	0.180 (0.218)
Obesity	0.181 (0.218)
Colorectal Neoplasms/pathology	0.181 (0.162)
Myocardial Infarction/diagnosis	0.182 (0.160)
Diabetes Mellitus/diagnosis	0.182 (0.201)
Acute Kidney Injury/blood	0.182 (0.216)
Muscle, Skeletal/physiopathology	0.184 (0.204)
Retreatment	0.184 (0.214)
Exercise Tolerance	0.184 (0.207)

Aortic Aneurysm, Thoracic/surgery	0.184 (0.237)
Breast Neoplasms/pathology	0.185 (0.141)
ST Elevation Myocardial Infarction/therapy	0.186 (0.231)
Outpatients	0.187 (0.133)
Hip Fractures/surgery	0.188 (0.157)
Developing Countries	0.190 (0.174)
Antibodies, Monoclonal, Humanized/adverse effects	0.190 (0.199)
Drug Administration Schedule	0.191 (0.095)*
Anticonvulsants/adverse effects	0.191 (0.215)
Self Efficacy	0.192 (0.179)
Schizophrenia/drug therapy	0.193 (0.240)
Residence Characteristics	0.194 (0.190)
Vitamin D Deficiency/blood	0.194 (0.281)
Administration, Oral	0.195 (0.096)*
Stomach Neoplasms/surgery	0.197 (0.223)
Laparoscopy/methods	0.197 (0.123)
Heart Failure/therapy	0.198 (0.124)
Postoperative Complications/therapy	0.198 (0.178)
Hematopoietic Stem Cell Transplantation/adverse effects	0.199 (0.203)
CD4 Lymphocyte Count	0.199 (0.171)
Lung Neoplasms/genetics	0.199 (0.390)
Breast Neoplasms/drug therapy	0.199 (0.139)
Referral and Consultation	0.199 (0.107)
Mental Disorders/epidemiology	0.201 (0.164)
Stress, Psychological/epidemiology	0.201 (0.217)
Respiration	0.202 (0.184)
Anti-HIV Agents/therapeutic use	0.205 (0.160)
Retinal Vessels/pathology	0.205 (0.210)
Colonoscopy	0.207 (0.172)
Weight Loss/physiology	0.207 (0.212)
Hemodynamics/physiology	0.207 (0.174)
Hypertension/epidemiology	0.210 (0.118)
Hospitals, University	0.212 (0.112)
Health Services Accessibility/statistics & numerical data	0.213 (0.205)
State Medicine	0.216 (0.179)
Vascular Surgical Procedures/adverse effects	0.217 (0.207)
Lung Neoplasms/pathology	0.217 (0.206)
Smoking/adverse effects	0.220 (0.152)
Carcinoma, Non-Small-Cell Lung/genetics	0.220 (0.435)
Area Under Curve	0.221 (0.124)
Thrombectomy/adverse effects	0.221 (0.243)
Cardiopulmonary Resuscitation/statistics & numerical data	0.222 (0.260)
Pandemics	0.222 (0.076)**
Psychometrics	0.223 (0.211)
Crohn Disease/complications	0.223 (0.227)
Pain/etiology	0.223 (0.145)
Obesity/physiopathology	0.223 (0.170)
Refraction, Ocular/physiology	0.225 (0.202)
Myocardial Infarction/drug therapy	0.226 (0.235)
Intensive Care Units/organization & administration	0.226 (0.174)
Hypertension/drug therapy	0.227 (0.162)
Hemoglobins/analysis	0.229 (0.163)
Imaging, Three-Dimensional	0.231 (0.175)
Diet	0.231 (0.130)
Genome-Wide Association Study	0.231 (0.225)

Observer Variation	0.232 (0.160)
Respiratory Distress Syndrome/therapy	0.232 (0.173)
Antibodies, Viral/blood	0.234 (0.226)
Patient Acceptance of Health Care/statistics & numerical data	0.234 (0.140)
Communicable Disease Control	0.235 (0.163)
Self Concept	0.235 (0.188)
Respiratory Insufficiency/therapy	0.237 (0.167)
Brain Ischemia/therapy	0.238 (0.255)
Dyslipidemias/drug therapy	0.239 (0.306)
Spondylitis, Ankylosing/drug therapy	0.239 (0.228)
Hyperglycemia/blood	0.240 (0.241)
Interleukin-6/blood	0.240 (0.180)
Triage	0.240 (0.159)
Animals	0.241 (0.101)*
Healthy Volunteers	0.241 (0.116)*
Lens Implantation, Intraocular	0.241 (0.249)
Pain Management	0.242 (0.199)
Forced Expiratory Volume	0.245 (0.165)
Pulmonary Disease, Chronic Obstructive/drug therapy	0.246 (0.193)
Hospitals, Teaching	0.248 (0.143)
Drug Therapy, Combination	0.248 (0.075)***
Diabetes Mellitus, Type 1/diagnosis	0.250 (0.279)
Renal Insufficiency, Chronic/etiology	0.251 (0.269)
Pulse Wave Analysis	0.251 (0.220)
Wounds and Injuries/mortality	0.252 (0.220)
Parathyroid Hormone/blood	0.253 (0.252)
Atherosclerosis/epidemiology	0.253 (0.261)
Dose-Response Relationship, Drug	0.253 (0.094)**
Lenses, Intraocular	0.255 (0.241)
Heart Atria/physiopathology	0.255 (0.290)
Combined Modality Therapy	0.255 (0.115)*
HIV Infections/prevention & control	0.256 (0.221)
Malnutrition/etiology	0.258 (0.246)
Cause of Death/trends	0.260 (0.208)
Infusions, Intravenous	0.260 (0.145)
Hemoglobins/metabolism	0.261 (0.171)
Punctures	0.262 (0.260)
Hypoglycemic Agents/adverse effects	0.262 (0.171)
Anti-Bacterial Agents/pharmacology	0.265 (0.156)
Limb Salvage	0.265 (0.291)
Decision Making	0.266 (0.145)
Quality of Life/psychology	0.266 (0.107)*
Stents/adverse effects	0.266 (0.216)
Mitral Valve/surgery	0.268 (0.247)
Coronary Vessels/diagnostic imaging	0.269 (0.176)
Recovery of Function	0.270 (0.080)***
Prosthesis Design	0.272 (0.110)*
Pulmonary Disease, Chronic Obstructive/mortality	0.272 (0.231)
Cardiac Catheterization/methods	0.274 (0.204)
Oxygen/blood	0.274 (0.143)
Health Knowledge, Attitudes, Practice	0.274 (0.107)*
Surgical Mesh	0.275 (0.214)
Preoperative Care	0.276 (0.188)
Endovascular Procedures/instrumentation	0.276 (0.240)
Heart Rate/drug effects	0.277 (0.220)

Cardiac Surgical Procedures/methods	0.277 (0.173)
Hospitals/statistics & numerical data	0.278 (0.202)
Intraocular Pressure/physiology	0.278 (0.170)
Acute Kidney Injury/therapy	0.279 (0.167)
Glucocorticoids/therapeutic use	0.280 (0.146)
Wound Healing	0.280 (0.177)
Guideline Adherence	0.280 (0.141)*
Myopia/surgery	0.280 (0.248)
Schools	0.280 (0.181)
Outcome Assessment, Health Care/statistics & numerical data	0.282 (0.204)
Computed Tomography Angiography/methods	0.282 (0.231)
Diabetes Mellitus/drug therapy	0.282 (0.234)
Carotid Stenosis/diagnostic imaging	0.283 (0.230)
Physical Therapy Modalities	0.283 (0.155)
Adenocarcinoma/pathology	0.283 (0.210)
Obesity/blood	0.284 (0.202)
Bone Density Conservation Agents/therapeutic use	0.285 (0.199)
Progression-Free Survival	0.286 (0.135)*
Death, Sudden, Cardiac/prevention & control	0.287 (0.282)
Stroke Rehabilitation	0.288 (0.186)
Bacteremia/microbiology	0.289 (0.218)
Remission Induction	0.290 (0.111)**
Outcome Assessment, Health Care	0.291 (0.083)***
Liver Neoplasms/surgery	0.291 (0.208)
Methotrexate/therapeutic use	0.292 (0.192)
Living Donors	0.293 (0.233)
Time-to-Treatment	0.293 (0.113)**
Clinical Decision-Making	0.295 (0.132)*
Respiratory Insufficiency/etiology	0.295 (0.227)
Contrast Media/administration & dosage	0.296 (0.232)
Brain/physiopathology	0.299 (0.232)
Pregnancy Rate	0.300 (0.165)
Oral Health	0.301 (0.193)
Angiography	0.302 (0.215)
Ambulatory Care Facilities	0.303 (0.186)
Psoriasis/drug therapy	0.306 (0.186)
Wounds and Injuries/epidemiology	0.306 (0.209)
Mitral Valve Insufficiency/physiopathology	0.308 (0.287)
Ambulatory Care	0.309 (0.173)
Liver Neoplasms/pathology	0.309 (0.341)
Communication	0.310 (0.129)*
Weight Loss	0.311 (0.120)**
Thrombolytic Therapy/methods	0.312 (0.223)
Carotid Arteries/diagnostic imaging	0.313 (0.271)
Fasting	0.313 (0.219)
Retinal Diseases/diagnosis	0.313 (0.221)
Stroke/prevention & control	0.313 (0.155)*
Renal Dialysis/methods	0.319 (0.193)
Waiting Lists	0.319 (0.218)
Operative Time	0.319 (0.115)**
Ultrasonography, Interventional/methods	0.320 (0.204)
Cholesterol, LDL/blood	0.322 (0.173)
Internship and Residency	0.326 (0.147)*
Cardiopulmonary Bypass	0.326 (0.215)
Liver Cirrhosis/mortality	0.327 (0.251)

Endoscopy	0.328 (0.192)
Administration, Intravenous	0.328 (0.137)*
Antineoplastic Agents/administration & dosage	0.333 (0.199)
Glaucoma/diagnosis	0.333 (0.239)
Caregivers/psychology	0.333 (0.165)*
Lumbar Vertebrae/diagnostic imaging	0.334 (0.167)*
Health Status Disparities	0.335 (0.177)
Anticonvulsants/therapeutic use	0.335 (0.168)*
Outcome and Process Assessment, Health Care	0.335 (0.163)*
Glycated Hemoglobin	0.337 (0.169)*
Acute Coronary Syndrome/blood	0.338 (0.233)
Electronic Health Records	0.340 (0.122)**
Tertiary Care Centers/statistics & numerical data	0.345 (0.160)*
Vascular Calcification/diagnostic imaging	0.345 (0.228)
Brain Ischemia/complications	0.346 (0.208)
Mitral Valve Insufficiency/surgery	0.346 (0.261)
Primary Health Care/methods	0.346 (0.222)
Insulin/blood	0.346 (0.204)
Insulin/administration & dosage	0.347 (0.230)
Referral and Consultation/statistics & numerical data	0.349 (0.157)*
Antiretroviral Therapy, Highly Active	0.350 (0.228)
Kidney Function Tests	0.350 (0.216)
Carcinoma, Non-Small-Cell Lung/mortality	0.351 (0.347)
Antibodies, Monoclonal, Humanized/therapeutic use	0.352 (0.120)**
Brain/metabolism	0.352 (0.241)
Postoperative Complications/surgery	0.352 (0.167)*
Coronavirus Infections/epidemiology	0.352 (0.463)
Coronary Vessels/physiopathology	0.353 (0.267)
Breast Feeding	0.354 (0.165)*
Hypothermia, Induced/methods	0.354 (0.228)
Aortic Valve Stenosis/mortality	0.358 (0.258)
Quality of Life	0.359 (0.039)***
Stents	0.364 (0.109)***
Staphylococcal Infections/epidemiology	0.365 (0.224)
Cardiac Catheterization/instrumentation	0.368 (0.287)
Multiple Sclerosis/drug therapy	0.370 (0.182)*
Aftercare	0.372 (0.194)
Dementia/epidemiology	0.372 (0.187)*
Myocardial Infarction/physiopathology	0.373 (0.231)
Mass Screening/methods	0.373 (0.167)*
Decision Support Techniques	0.375 (0.139)**
COVID-19 Vaccines	0.375 (0.186)*
Pain, Postoperative/drug therapy	0.375 (0.158)*
Drug Resistance	0.377 (0.195)
Triage/methods	0.377 (0.189)*
Protein Kinase Inhibitors/therapeutic use	0.378 (0.197)
Emergency Service, Hospital/organization & administration	0.382 (0.144)**
Diabetic Nephropathies/epidemiology	0.384 (0.270)
Monitoring, Intraoperative/methods	0.384 (0.196)*
Ventricular Dysfunction, Left/etiology	0.385 (0.284)
Patient Satisfaction	0.386 (0.085)***
Shock, Septic/therapy	0.387 (0.240)
Heart Valve Prosthesis	0.388 (0.206)
Blood Pressure/drug effects	0.390 (0.167)*
Disease Management	0.390 (0.141)**

Robotic Surgical Procedures/methods	0.391 (0.205)
Antifungal Agents/therapeutic use	0.392 (0.190)*
Vascular Patency	0.393 (0.176)*
Coronary Artery Bypass/mortality	0.397 (0.237)
Models, Biological	0.399 (0.204)
COVID-19 Testing	0.400 (0.189)*
Posture/physiology	0.400 (0.206)
COVID-19/prevention & control	0.402 (0.107)***
Pulmonary Disease, Chronic Obstructive/therapy	0.406 (0.189)*
Death, Sudden, Cardiac/etiology	0.409 (0.283)
Blood Pressure Monitoring, Ambulatory	0.410 (0.234)
Calcium/blood	0.411 (0.240)
Drug Substitution	0.416 (0.185)*
Alleles	0.416 (0.236)
Obesity, Morbid/complications	0.417 (0.212)*
Transcatheter Aortic Valve Replacement/methods	0.417 (0.242)
Kidney/physiopathology	0.418 (0.155)**
Quality-Adjusted Life Years	0.418 (0.224)
Bayes Theorem	0.418 (0.186)*
Life Style	0.419 (0.133)**
Plastic Surgery Procedures/methods	0.420 (0.199)*
Vascular Endothelial Growth Factor A/antagonists & inhibitors	0.420 (0.265)
Metabolic Syndrome/diagnosis	0.420 (0.273)
Dyslipidemias/diagnosis	0.422 (0.369)
Antineoplastic Combined Chemotherapy Protocols/adverse effects	0.424 (0.153)**
Wet Macular Degeneration/diagnosis	0.425 (0.300)
Pneumonia/mortality	0.427 (0.249)
Natriuretic Peptide, Brain/blood	0.428 (0.207)*
Knee Joint/diagnostic imaging	0.429 (0.234)
Body Height	0.433 (0.201)*
Vaccination	0.436 (0.140)**
Point-of-Care Systems	0.438 (0.157)**
Sodium-Glucose Transporter 2 Inhibitors/therapeutic use	0.440 (0.215)*
Patient Safety	0.445 (0.132)***
Cardiovascular Diseases/prevention & control	0.445 (0.151)**
Recombinant Proteins/therapeutic use	0.449 (0.202)*
Clinical Competence	0.455 (0.106)***
Students, Medical	0.458 (0.207)*
Hepatitis C, Chronic/drug therapy	0.464 (0.217)*
Mendelian Randomization Analysis	0.467 (0.186)*
Hemorrhage/prevention & control	0.471 (0.230)*
Postoperative Complications/prevention & control	0.473 (0.115)***
Diabetes Mellitus, Type 1/drug therapy	0.473 (0.186)*
Drainage/methods	0.477 (0.210)*
Neurosurgical Procedures/methods	0.477 (0.206)*
Diabetes Mellitus/therapy	0.479 (0.234)*
Pain Management/methods	0.480 (0.141)***
Prostatic Neoplasms/diagnosis	0.483 (0.238)*
Catheter Ablation/methods	0.484 (0.171)**
Enteral Nutrition/methods	0.485 (0.212)*
Pain/drug therapy	0.486 (0.204)*
Cardiovascular Diseases/blood	0.491 (0.170)**
Cystic Fibrosis/drug therapy	0.495 (0.222)*
Lipids/blood	0.496 (0.161)**
Dietary Supplements	0.497 (0.163)**

Treatment Outcome	0.499 (0.023)***
Testosterone/blood	0.503 (0.201)*
Sleep Apnea, Obstructive/therapy	0.506 (0.242)*
Coronary Stenosis/diagnostic imaging	0.509 (0.214)*
Health Services Accessibility	0.515 (0.169)**
Research Design	0.519 (0.198)**
Algorithms	0.537 (0.103)***
Mass Screening	0.540 (0.196)**
Inflammation Mediators/blood	0.546 (0.209)**
Neoplasm Recurrence, Local/drug therapy	0.550 (0.216)*
Minimally Invasive Surgical Procedures/methods	0.558 (0.208)**
Artificial Intelligence	0.560 (0.200)**
Immunosuppressive Agents/administration & dosage	0.564 (0.202)**
Glucocorticoids/administration & dosage	0.565 (0.181)**
Early Detection of Cancer/methods	0.565 (0.204)**
Risk Reduction Behavior	0.565 (0.202)**
Mobile Applications	0.571 (0.207)**
Fracture Fixation, Internal/methods	0.573 (0.196)**
Electronic Health Records/statistics & numerical data	0.577 (0.216)**
BNT162 Vaccine	0.583 (0.231)*
Feasibility Studies	0.591 (0.080)***
Corneal Topography	0.612 (0.213)**
Equipment Design	0.621 (0.110)***
Colitis, Ulcerative/drug therapy	0.625 (0.177)***
Curriculum	0.633 (0.196)**
Multiple Sclerosis, Relapsing-Remitting/drug therapy	0.645 (0.173)***
Ultrasonography, Interventional	0.648 (0.157)***
Clinical Protocols	0.653 (0.174)***
Antibodies, Monoclonal/therapeutic use	0.655 (0.160)***
Nerve Block/methods	0.661 (0.240)**
Disease Outbreaks	0.686 (0.187)***
Venous Thromboembolism/prevention & control	0.699 (0.249)**
Qualitative Research	0.703 (0.161)***
Intravitreal Injections	0.721 (0.198)***
Surgical Wound Infection/prevention & control	0.731 (0.212)***
Telemedicine	0.732 (0.164)***
Embolization, Therapeutic/methods	0.735 (0.209)***
Vitrectomy/methods	0.765 (0.196)***
Pain, Postoperative/prevention & control	0.769 (0.226)***
Pandemics/prevention & control	0.779 (0.219)***
Anti-Asthmatic Agents/therapeutic use	0.795 (0.257)**
Patient Care Team	0.816 (0.179)***
Cross Infection/prevention & control	0.837 (0.197)***
Quality Improvement	0.857 (0.121)***
Drug Combinations	0.861 (0.115)***
Mice	0.901 (0.214)***
Exercise Therapy/methods	0.947 (0.190)***
Sustained Virologic Response	0.970 (0.225)***
Cost-Benefit Analysis	0.990 (0.123)***
Pharmacists	1.014 (0.196)***
Product Surveillance, Postmarketing	1.042 (0.215)***
Radiation Dosage	1.045 (0.199)***
Program Evaluation	1.247 (0.162)***
